# Regulating the cell differentiation trajectory of progenitor cells in adipose tissue fibrosis

**DOI:** 10.1016/j.molmet.2025.102231

**Published:** 2025-08-06

**Authors:** Li Zhang, Xinjiang Cai, Xiuju Wu, Zheng Jing, Yan Zhao, Yucheng Yao, Kristina I. Boström

**Affiliations:** 1 Division of Cardiology, David Geffen School of Medicine at UCLA, USA; 2 Molecular Biology Institute, University of California, Los Angeles, Los Angeles, CA, USA

**Keywords:** White adipose tissue, Matrix Gla protein, Dipeptidyl peptidase-4, Adipose progenitor cell differentiation, Adipose fibrosis, Transforming growth factor β signaling, Bone morphogenetic protein, Single-cell RNA sequencing

## Abstract

**Objective:**

Adipose fibrosis signifies pathological remodeling of white adipose tissue (WAT) associated with insulin resistance, diabetes, and cardiovascular disease. Matrix Gla protein (MGP) balances bone morphogenetic protein (BMP) and transforming growth factor β (TGFβ) signaling but has an unclear role in WAT.

**Methods:**

To study the role of MGP in WAT, we used mice with global or platelet-derived growth factor receptor α (*Pdgfra*)-Cre-mediated *Mgp* deletion in adipose progenitor cells (APCs) together with single cell RNA sequencing (scRNA-seq), characterization on adipose and fibrotic phenotypes, and BMP and TGFβ signaling studies.

**Results:**

Our results showed that Mgp deletion promotes fibrosis and impairs adipogenesis in mice with global or *Pdgfra*-Cre-mediated *Mgp* deletion in APCs. ScRNA-seq showed two new adipose-derived stem cells (ASC) populations, ASC1 and ASC4, emerging after *Mgp* deletion. Trajectory analysis found that ASC1 and ASC4 were derived from ASC2, which normally undergo adipogenesis but instead had diverted to fibrogenic differentiation. All three ASCs expressed *Pdgfra* and dipeptidyl peptidase-4 (*Dpp4*). Inhibition of TGFβ signaling or DPP4 activity in mice with *Pdgfra*-Cre-mediated *Mgp* deletion reduced the size of the PDGFRα+; DPP4+ cell population and rescued the WAT from unwanted fibrosis.

**Conclusions:**

MGP is essential for appropriate balance between adipogenic differentiation and fibroblast activation. Dysregulation of PDGFRα+; DPP4+ cells may signal early adipose fibrosis.

## Highlights

• *Mgp* gene deletion promotes adipose fibrosis and impairs white adipogenesis in mice.
• Two new PDGFRα+; DPP4+ cell clusters emerge after *Mgp* deletion.
• The PDGFRα+; DPP4+ cell clusters are limited in size by inhibition of DPP4 or ALK5.
• Adipose fibrosis due to *Mgp* deletion is rescued by inhibition of DPP4 or ALK5, but worsens with high fat feeding.

## 1 Introduction

Fibrosis is defined as the scarring of tissue when pathological repair, metabolic changes or disease trigger a maladaptive fibrogenic process [1]. Adipose fibrosis refers to the excessive tissue deposition of extracellular matrix (ECM) components, such as collagens, elastin and fibronectin, and may be driven by chronic inflammation, adipocyte dysfunction, fibroblast activation and ECM remodeling [[2], [3], [4], [5], [6]]. Fibrosis impairs the expandability and function of adipose tissue, leading to ectopic fat deposition and systemic insulin resistance [7].

Adipose progenitor cells (APCs) are found in adipose tissue, where they undergo commitment to preadipocytes and have the potential to ultimately differentiate into mature adipocytes. Although the APCs are critical for growth and maintenance of adipose tissue, they may also contribute to the development of adipose fibrosis under pathological conditions. The APCs are heterogenous and exist as distinct cell populations in the stromal vascular fraction (SVF). Platelet-derived growth factor receptor α (PDGFRα) is a cell surface receptor tyrosine kinase that has a role in both adipogenic and fibrogenic differentiation in adipose tissue. Berry & Rodeheffer [8] showed that a PDGFRα trace labeled all the white adipocytes, and specifically identified Lin-: CD29+, CD34+, Sca-1+, CD24+ (CD24+) cells and Lin+: CD29+, CD34+, Sca1+, CD24- (CD24-) cells, as adipocyte precursors. Shin et al. [9] showed that PDGFRα+ cells are adipogenic during development, and that *Pdgfra* gene deletion disrupts white adipose tissue (WAT) formation and cause embryonic APCs to shift to fibrogenic lineage. *Pdgfra* is also highly expressed in fibroblasts and its activation leads to transition into myofibroblasts, which are key drivers of ECM production and fibrosis [10]. Marcelin et al. [11] showed that CD9 expression distinguished two subsets of adipose PDGFRα+ APCs, where high CD9 expression is pro-fibrogenic and drives adipose fibrosis.

Recently, single-cell RNA sequencing (scRNA-seq) identified a new, highly proliferative adipose progenitor population that express dipeptidyl peptidase-4 (DPP4+), a serine protease implicated in both adipogenesis and fibrosis. In healthy adipose tissue, DPP4+ cells differentiate to preadipocytes (ICAM1+) [12], which subsequently give rise to adipocytes. In addition, DPP4 is highly expressed on the surface of fibroblasts where it promotes the transition to myofibroblasts, and is considered one of the markers of activated fibroblasts in systemic sclerosis [13]. Several studies have shown elevated *Dpp4* expression in fibrosis organs such as liver, kidneys, lungs, and heart [[14], [15], [16], [17], [18], [19]].

Matrix Gla protein (MGP) is a small, secreted protein, which serves as a regulator of bone morphogenetic protein (BMP) activity and subsequent transforming growth factor β (TGFβ) signaling [[20], [21], [22]]. MGP plays an important role in the regulation of ectopic osteogenesis, vascular morphogenesis, and arterial calcification [[23], [24], [25], [26]]. In addition, it mediates control of intermediate steps involving BMP4 and BMP7 in brown adipose tissue (BAT) differentiation [27]. Considering that BMPs are also important in WAT [[28], [29], [30]] and TGFβ signaling promotes fibrosis [[31], [32], [33]] while obstructing adipogenesis [34], we hypothesized that MGP regulates the fate of APCs by balancing BMP and TGFβ signaling.

In this study, we investigated the role of MGP in white adipogenesis and adipose fibrosis. We found that both global (*Mgp*-KO) and *Pdgfrα Cre*-mediated *Mgp* deletion (*Pd*–KO) in mice increased the PDGFRα+; DPP4+ cell population, inhibited adipogenesis and promoted fibrosis. ScRNA-seq revealed a new APC trajectory, which diverted adipogenic to fibrogenic differentiation with increased PDGFRα+; DPP4+ cell populations and TGFβ components.

Reduction of TGFβ signaling, DPP4 activity or the PDGFRα+; DPP4+ cell population rescued the adipose tissue from unwanted fibrosis. Thus, MGP is essential for the balance between adipogenic differentiation and fibroblast activation.

## 2 Results

### 2.1 Increased fibrosis and decreased adipogenesis in WAT of global matrix Gla protein (Mgp) knockout (KO) mice

**Figure 1:**
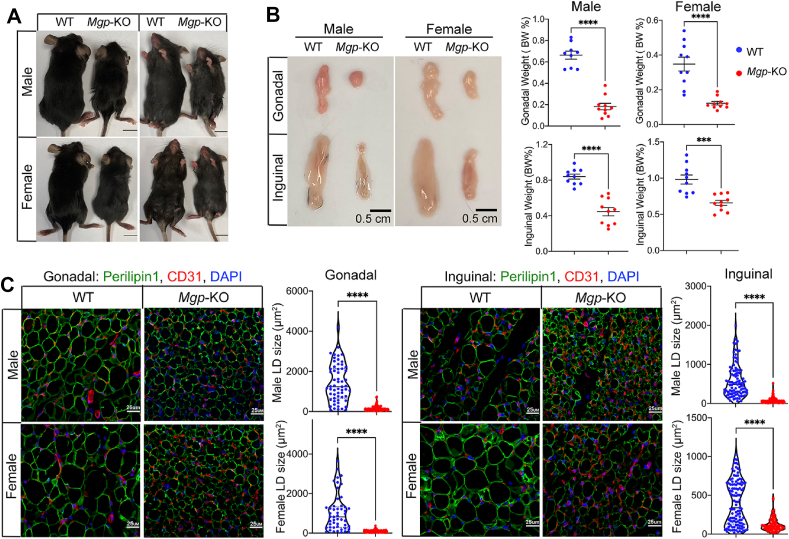
Global matrix Gla protein (Mgp) deletion in C57BL/6 mice causes a reduction in white adipose development. (A) Wild-type (WT) and *Mgp*-knockout (*Mgp-*KO) male and female mice at 4 weeks of age. Scale bars, 1 cm. (B) Gonadal and inguinal fat pads from WT and *Mgp-*KO male and female mice at 4 weeks of age, normalized to body weight (right), *n* = 10. Scale bars 0.5 cm. (C) Immunofluorescence for Perilipin-1 (green, for lipid droplet (LD) size) and CD31 (red) in gonadal (left) and inguinal (right) white adipose tissue from WT and *Mgp-*KO mice at 4 weeks of age. DAPI (blue) was used to visualize nuclei. Scale bars, 25 μm. The LD size was determined by ImageJ (*n* = 5 mice, 3–4 fields of view per replicate). Data are shown as mean ± SEM; One way ANOVA with Tukey’s post hoc tests; ∗∗∗*p* < 0.001, ∗∗∗∗*p* < 0.0001.

Our previous studies showed that *Mgp*-KO mice have a reduction in body weight and percent fat, starting at two weeks of age in both male and female mice [27]. We also showed that lack of MGP disrupts BMP-regulated steps in brown adipogenesis [27]. However, the effect on development and progenitor cells in white adipose tissue (WAT), the main adipose component, has not been clarified. Therefore, we examined subcutaneous and visceral adipose tissue, represented by inguinal and gonadal fat pads, respectively. We found that the fat pads were smaller in both male and female *Mgp*-KO mice, with significant reductions in mass when normalized to body weight (Figure 1A,B). The lipid accumulation in the adipocyte was outlined by immunofluorescence for Perilipin-1 and the area was quantified by Image J. The results revealed a reduction in lipid droplet size in inguinal and gonadal fat pads of both genders compared to wild-type (WT) (Figure 1C). Together, the findings suggested that lack of MGP also disrupted the WAT development, potentially involving fibrogenic activation. Since the male and female phenotypes were similar, the genders were subsequently analyzed together.

**Figure 2:**
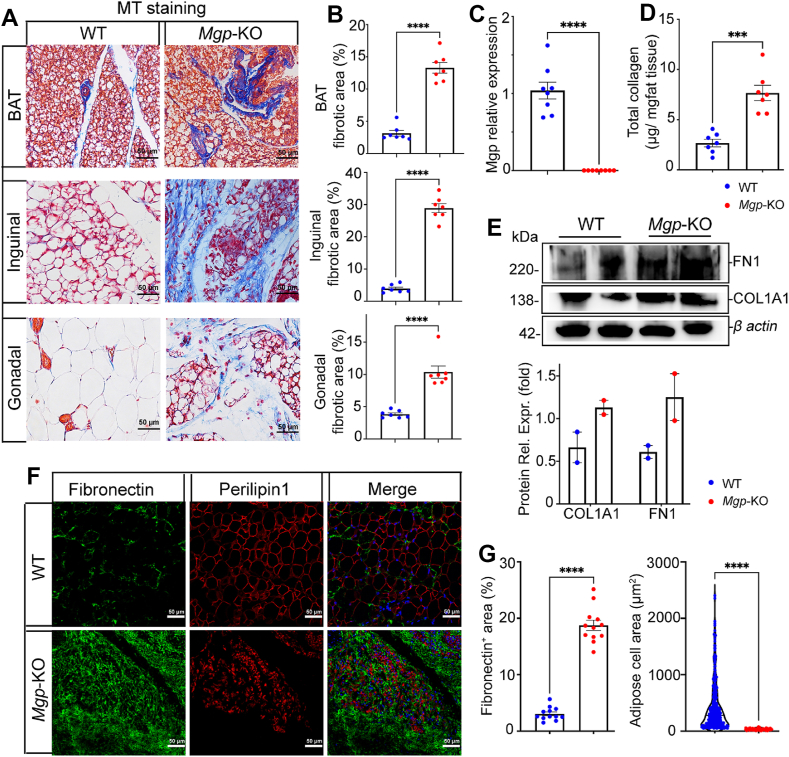
Global Mgp deletion in mice enhances adipose fibrosis. (A) Masson’s trichrome (MT) staining of adipose tissue from wild-type (WT) and *Mgp*-knockout (*Mgp-*KO) mice at 4 weeks. Scale bars, 50 μm. (B) Quantification of fibrotic (blue) MT staining in WT and *Mgp-*KO mice at 4 weeks of age demonstrating increased fibrosis after *Mgp* deletion, as determined by ImageJ (*n* = 3 mice, 7–8 fields of view per replicate). (C) *Mgp* expression in inguinal adipose tissue from WT and *Mgp-*KO mice at 4 weeks, as determined by qPCR (*n* = 8). (D) Total collagen in inguinal adipose tissue from WT and *Mgp-*KO mice at 4 weeks, as determined by the free hydroxyproline method (*n* = 7). (E) Expression of Fibronectin (FN1) and collagen type I alpha-1 chain (COL1A1) as determined by immunoblotting with densitometry (representative of 3 independent experiments, mean of 2 lanes per replicate); β-actin was used as control. (F) Immunofluorescence for Fibronectin (green) and Perilipin-1 (red) in inguinal adipose tissue from WT and *Mgp-*KO mice at 4 weeks. DAPI was used to visualize the nuclei. Scale bars, 50 μm (*n* = 3). (G) Quantification of area stained for Fibronectin in WT and *Mgp-*KO mice at 4 weeks, as determined by ImageJ (*n* = 3, 10–15 fields of view per replicate). Data are shown as mean ± SEM; One way ANOVA with Tukey’s post hoc tests; ∗∗∗∗*p* < 0.0001.

Adipose fibrosis represents a pathological remodeling of adipose tissue characterized by excessive deposition of collagen fibers and ECM. Indeed, Masson’s trichrome (MT) staining revealed dramatic increases in areas stained blue for collagen in all the fat pads of the *Mgp*-KO mice, including inguinal, gonadal, and brown adipose tissue (Figure 2A, Supplemental Fig. S1). The largest increase in collagen-rich area was observed in the inguinal fat pads (Figure 2A,B). Therefore, we focused on the inguinal adipose tissue in subsequent analyses. The lack of *Mgp* expression in the inguinal fat was confirmed by qPCR (Figure 2C). Total collagen significantly increased in *Mgp*-KO mice compared to wild-type controls, as determined by the release of free hydroxyproline by alkaline hydrolysis (Figure 2D). Furthermore, the expression of collagen type 1 alpha 1 (Col1A1) and fibronectin was increased as determined by immunoblotting with densitometry (Figure 2E). Immunofluorescence also showed a striking increase in fibronectin in *Mgp*-KO adipose tissue in combination with a decrease in lipid droplet size by Perilipin-1 staining (Figure 2F,G). Thus, the results suggested that global loss of *Mgp* promotes fibrosis at the expense of adipogenesis.

### 2.2 Single-cell RNA sequencing (scRNA-seq) revealed two new subclusters of progenitor cells in the SVF from Mgp-KO mice

**Figure 3:**
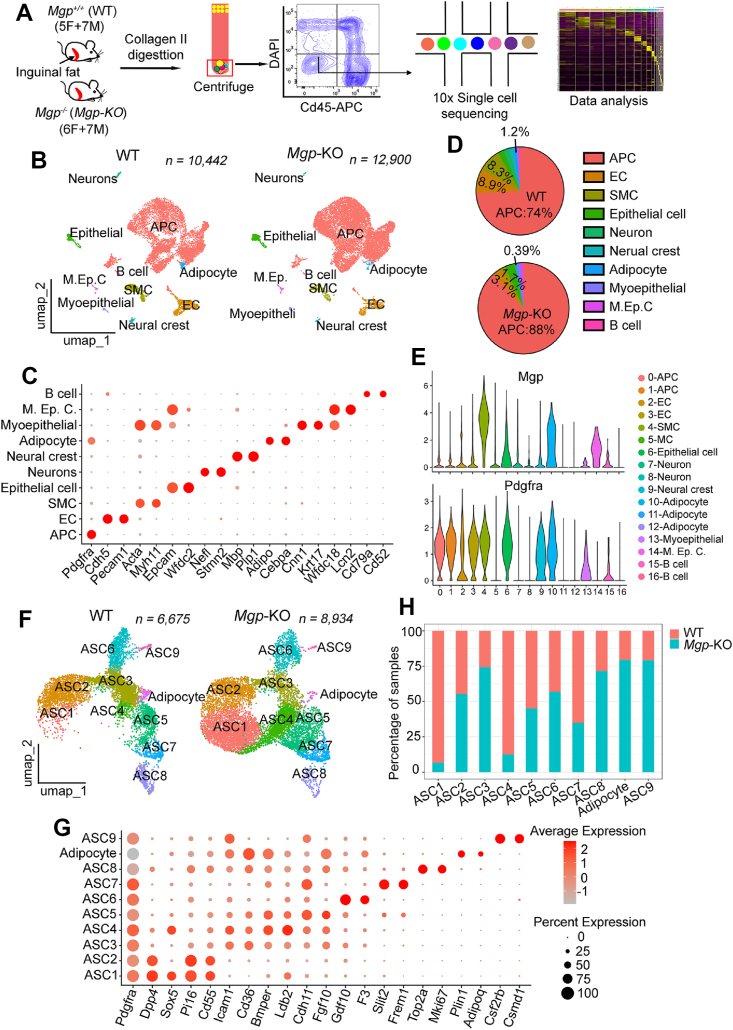
Single cell RNA-seq analysis (scRNA-seq) reveals the altered composition of the stromal vascular fraction (SVF) from inguinal adipose tissue from wild-type (WT) versus *Mgp*-knockout (*Mgp*-KO) mice. A. Schematic outline of the experimental pipeline. The SVF from white inguinal adipose tissue was collected from 4-week-old WT mice (5 female and 7 male) and *Mgp-*KO mice (6 female and 7 male). CD45 negative stromal vascular fraction (SVF) were isolated, subjected to scRNA-seq and clustered using Seurat. B. Uniform Manifold Approximation and Projection (UMAP) plot of 23,342 SVF cells from WT and *Mgp-*KO mice separated into 17 distinct clusters and 10 cell types. C. DotPlot showing the marker genes for each cluster. D. Percent of each cell type in SVF from WT and *Mgp*-KO adipose tissue. E. VlnPlot showing *Pdgfrα* and *Mgp* expression in each cluster. The results show *Mgp* expression mainly in *Pdgfrα*-positive cell clusters. F. Re-clustering of 15,609 *Pdgfrα*-positive adipose progenitor cells and adipocytes. Ten cell clusters were identified, with almost complete absence of ASC1 and ASC4 in *WT* adipose tissue. G. Marker genes of each cell type (DotPlot). H. Percent of each cell type in adipose tissue from WT (blue) and *Mgp*-KO (red) mice (ggplot).

To investigate potential changes in the cellular composition of the SVF after loss of *Mgp*, we obtained inguinal fat pads from 12 wild-type mice (5 female, 7 male) and 13 global *Mgp*-KO mice (6 female, 7 male). Following SVF isolation, lipid-loaded adipocytes and CD45+ immune cells were removed through centrifugation and FACS. Samples were subjected to scRNA-seq using 10x Genomics (Figure 3A). After stringent quality filtering, we obtained transcriptomes of 23,342 cells (10,442 wild-type and 12,900 *Mgp*-KO cells). Unbiased clustering resulted in 17 clusters (Supplemental Fig. S2a). Based on established lineage-specific markers, we assigned these clusters to 10 cell types (Figure 3B). These included adipose progenitor cells (APCs; identified by *Pdgfra* and *Dcn*) [35], early adipocytes (*Adipoq* and *Cebpa*) [36], endothelial cells (ECs, *Cdh5* and *Pecam1*) [37], smooth muscle cells (SMCs; *Acta2* and *Myh11*) [38], myoepithelial cells (MECs; *Cnn1* and *Krt17*) [39], epithelial cells (*Epcam* and *Wfdc2*) [40], neural crest-derived cells (*Mbp and Plp1*), and neurons (*Nefl* and *Stmn2*) [12], together representing the main components of the SVF (Figure 3C).

The APCs were the predominant cell population, and were found in clusters 0–4, 6, and 8–9 (Supplemental Fig. S2b). These clusters accounted for 74% and 88% of the cells in the wild-type and *Mgp*-KO SVFs, respectively (Figure 3D). *Pdgfra*-expressing cells included all of the previously identified APCs, such as the experimentally tested APCs with the surface markers CD29+, CD34+, LY6A+, and CD24- [8], as well as more recently identified interstitial progenitor cells (DPP4+), committed preadipocytes (ICAM1+) and adipogenesis regulatory cells (Aregs, F3+) [12] (Supplemental Fig. S2b). To further analyze the transition from APCs to adipocytes, we studied the *Pdgfra*-expressing cells in the wild-type scRNA-seq data, where *Mgp* was highly expressed (Figure 3E). *Pdgfra-*expressing cells (with normalized gene expression >0.1) and the adipocyte fraction were subsetted; a total of 15,609 cells contained 6,675 wild-type and 8,934 *Mgp*-KO cells. Re-clustering of these cells separated them into 10 clusters, 9 of which were referred to as ASC1-9 with *Pdgfra*-expression, whereas the remaining cluster contained early adipocytes (Figure 3F,G). Interestingly, the ASC1 and ASC4 clusters were noted predominantly in the *Mgp*-KO mice, where the adipocyte population was reduced (Figure 3H). Thus, the scRNA-seq analysis supported that *Mgp* deletion limited APCs undergoing adipogenic differentiation and thereby increased the availability of APCs for fibrogenesis. It is possible that adipocytes and fibrogenic cells originate from the same *Pdgfra-*expressing progenitor cells, in line with previous proposals that MGP is an important factor in determining adipogenic versus fibrogenic differentiation.

### 2.3 Pdgfrα Cre-mediated Mgp-deletion decreased adipogenesis and increased fibrosis

**Figure 4:**
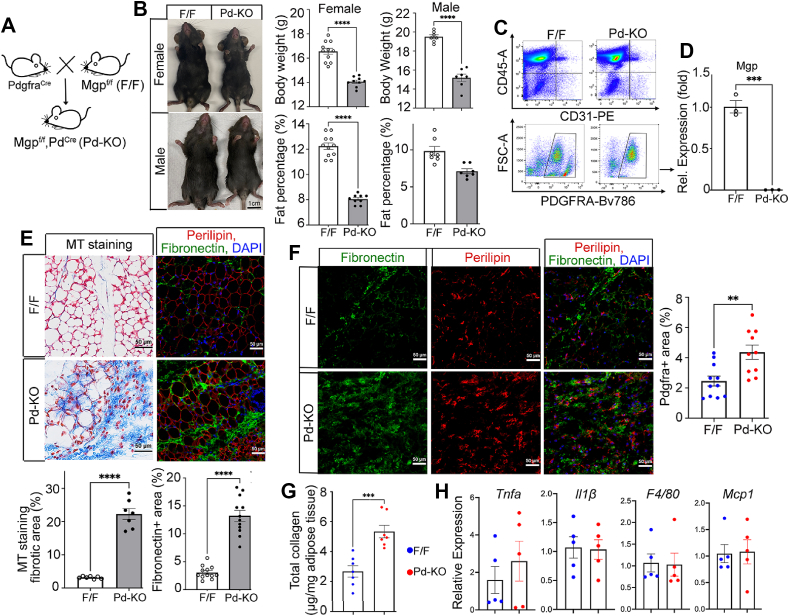
Pdgfrα Cre-guided Mgp deletion enhance adipose fibrosis. A. Schematic outline of breeding strategy for *Mgp*^f/f^;*Pd*^Cre^ (*Pd*–KO) mice. B. Body weight and percent fat of male and female *Mgp*^f/f^ (F/F), and *Pd*–KO mice at 4 weeks (*n* = 7–11). Scale bar, 1 cm. C. FACS analysis of PDGFRα+ cells from CD45-, CD31-live cells from F/F and *Pd*–KO mice (*n* = 5 mice per group). D. *Mgp* expression in *Pdgfrα*-expressing cells isolated from F/F and *Pd*–KO mice at 4 weeks (*n* = 3–5 mice per group, 3 replicates). E. (Top) Masson’s trichrome (MT) staining and immunofluorescence for Perilipin-1 (red), Fibronectin (green) and DAPI (blue) in inguinal adipose tissue from F/F and *Pd*–KO mice at 4 weeks. (Bottom) Quantification of fibrotic (blue) MT staining and Fibronectin immunostaining from F/F and *Pd*–KO mice at 4 weeks demonstrating increased fibrosis in the *Pd*–KO mice (*n* = 3 mice, 3–4 fields of view per replicate). F. (Right) Immunofluorescence for Fibronectin (green) and PDGFRα (red) in inguinal adipose tissue from F/F and *Pd*–KO mice at 4 weeks. DAPI (blue) was used to visualize the nuclei. Bars, 50 μm (*n* = 3). (Left) Percent area stained for PDGFRα in F/F and *Pd*–KO mice at 4 weeks, as determined by ImageJ (*n* = 3 mice, 3–4 fields of view per replicate). G. Total collagen normalized to adipose tissue in inguinal adipose tissue from F/F and *Pd*–KO mice at 4 weeks, as determined by the free hydroxyproline method (*n* = 7 independent samples). H. Gene expression of *Tnfa* (Tumor Necrosis Factor), *Il1β* (Interleukin 1β)*,* F4/80 (also known as *Adgre1,* a macrophage marker), and *Mcp1* (Monocyte chemoattractant protein-1, also known as *Ccl2*) in inguinal adipose tissue from F/F and *Pd*–KO mice at 4 weeks as determined by qPCR (*n* = 5 mice per group). Data are shown as mean ± SEM; One way ANOVA with Tukey’s post hoc tests; ∗∗*p* < 0.01, ∗∗∗*p* < 0.001, ∗∗∗∗*p* < 0.0001.

To test the hypothesis that *Mgp* deletion in *Pdgfrα*-expressing APCs enhanced fibrosis, we took advantage of the observation that more than 85% of the *Mgp-*expressing cells expressed *Pdgfrα* (Figure 3E). We deleted *Mgp* specifically in *Pdgfra+* cells using the *Mgp*-floxed (*Mgp*^*flox/flox*^, F/F) mice crossbred with the *Pdgfra-Cre* transgenic mice to generate *Mgp*^*flox/flox*^;*Pdgfrα*^*Cre*^ (*Pd*–KO) mice (Figure 4A), using the F/F mice as controls. Lack of *Mgp* expression was confirmed in the sorted PDGFRα+ cells from the *Pd*–KO mice (Figure 4C,D). NMR assessment showed a significant reduction in body weight and percent fat in both male and female *Pd*–KO mice at 4 weeks of age (Figure 4B). In addition, the fat pad weight and lipid droplet size decreased whereas the vascular density, as determined by immunostaining for CD31, increased in both inguinal and gonadal adipose tissue in the *Pd*–KO mice compared to F/F controls (Supplemental Fig. S3a–c).

We examined the adipose tissue for fibrosis, and found significantly larger areas of collagen in the *Pd*–KO mice compared to controls, based on MT staining (Figure 4E). The *Pd*–KO mice also had larger areas of positive fibronectin-staining, especially around the adipocytes (Figure 4E). These positive areas showed enhanced expression of *Pdgfra* in the *Pd*–KO mice compared to controls (Figure 4F). In addition, the total collagen content was significantly increased in the *Pd*–KO mice (Figure 4G), suggesting that the ECM deposition was enhanced in the *Pd*–KO mice. To assess if the fibrosis was associated with inflammation, we examined several markers in inguinal adipose tissue from 4-week-old *Pd*–KO and F/F mice by qPCR. The results showed that expression of *Il-1* and *Il-6* was undetermined, and expression of *Tnf, Il1β, Mcp1* and the macrophage marker *F4/80* did not differ between *Pd*–KO and controls (Figure 4H). It suggested that the fibrosis was not caused by inflammation, but that deletion of *Mgp* in progenitor cells diverted differentiation toward fibrogenesis in the *Pd*–KO mice at a young age. It is possible that inflammation in adults take advantage of this pathway to trigger fibrosis.

### 2.4 Pdgfra-Cre guided Mgp deletion enhanced adipose fibrosis in HFD

**Figure 5:**
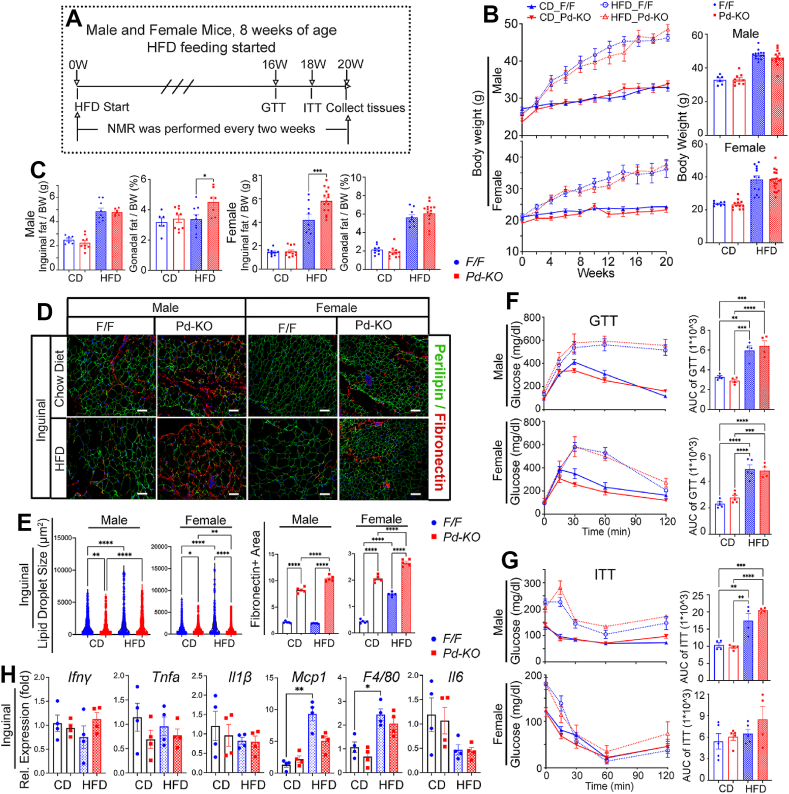
Pdgfra-Cre-guided Mgp deletion enhances adipose fibrosis after high fat feeding. A. Experimental timeline for feeding of *Mgp*^f/f^ (F/F) and *Mgp*^f/f^,*Pd*^Cre^ (*Pd*–KO) mice with chow diet (CD) or 40% high fat diet (HFD), starting at 8 weeks of age. W, weeks. B. Left panels: comparison of body weights between F/F and *Pd*–KO mice on either CD or HFD (*n* = 8–21 per group) every two weeks. Right panels: final body weights of female and male F/F and *Pd*–KO mice after 20 weeks of CD and HFD, as determined by NMR. C. Percent inguinal and gonadal fat of male and female F/F and *Pd*–KO mice at 28 weeks (*n* = 8–21). D. Immunofluorescence for Perilipin-1 (green), Fibronectin (red) in inguinal adipose tissue from F/F and *Pd*–KO mice at 28 weeks. DAPI (blue) was used to visualize the nuclei. Bars, 50 μm (*n* = 5, representative of 3–4 fields of view per replicate). E. Quantification of Fibronectin positive area and lipid droplet size in Panel d from F/F and *Pd*–KO mice at 28 weeks (*n* = 5, 3–4 fields of view per replicate). F. Glucose tolerance test (GTT) at 16 weeks in F/F and *Pd*–KO mice after CD and HFD. (Right) AUC, GTT area under the curve, (*n* = 4–5). G. Insulin tolerance test (ITT) at 18 weeks in F/F and *Pd*–KO mice after CD and HFD. (Right) AUC, ITT area under the curve, (*n* = 4–5). H. Gene expression of *Ifnγ* (Interferon γ), *Tnfa* (Tumor Necrosis Factor), *Il1β* (Interleukin 1β)*, Mcp1* (Monocyte chemoattractant protein-1, also known as *Ccl2*)*, F4/80* (also known as *Adgre1,* a macrophage marker), and *Il6* (Interleukin 6) in inguinal adipose tissue from F/F and *Pd*–KO mice at 28 weeks as determined by qPCR (*n* = 4 per group). Data are shown as mean ± SEM; One way ANOVA with Tukey’s post hoc tests; ∗*p* < 0.05, ∗∗*p* < 0.01, ∗∗∗*p* < 0.001, ∗∗∗∗*p* < 0.0001.

To examine if deletion of *Mgp* in *Pdgfra*-expressing progenitors affects diet-induced obesity in adult mice, we treated male and female *Pd*–KO and F/F control mice for 20 weeks with chow diet (CD) or high-fat diet (HFD; 45 kcal% fat) starting at 8 weeks of age (Figure 5A for timeline). At 8 weeks of age, prior to HFD, there was no significant difference in body weight between *Pd*–KO and control mice of both genders (Figure 5B). After 20 weeks on HFD, body weight and size of fat pads normalized to body weight increased compared to CD, but there was no significant difference between the *Pd*–KO mice and controls on either diet, with the exception of larger inguinal fat pads in females on HFD (Figure 5B,C).

In the inguinal fat, the size of the lipid droplets was reduced in *Pd*–KO mice on both diets and in both genders, as outlined by Perilipin-1 staining (Figure 5D,E). In addition, *Pd*–KO mice of both genders exhibited increased serum non-esterified fatty acid (NEFA) levels compared to F/F controls on CD, but not on HFD (Supplemental Fig. S4a). Serum triglycerides (L-TG) were similar in *Pd*–KO and F/F mice on the same diet (Supplemental Fig. S4a). To further investigate whether *Mgp* deletion in the adipose progenitors would affect the metabolic phenotype, we performed glucose tolerance tests (GTT) and insulin tolerance test (ITT) in *Pd*–KO and F/F mice of both genders on HFD or CD. No significant difference was detected in the AUC of the GTT or the ITT between the *Pd*–KO and F/F control mice on the same diet (Figure 5F,G).

However, the fibronectin-positive areas increased significantly in the fat pads of both *Pd*–KO mice and controls after HFD, especially in the inguinal fat pads of male *Pd*–KO mice (Figure 5D,E). To test if increased inflammation contributed to the fibrosis, we checked several inflammatory markers. Inguinal expression of *Ifnγ*, *Tnfα*, *Il1β* and *Il6* did not differ between *Pd*–KO and F/F mice on the same diet (Figure 5H). However, expression of *Mcp1* and *Adgre1* (F4/80) was increased in *Pd*–KO and F/F mice on HFD (Figure 5H), and may promote the development of fibrosis. Together, the results suggest that the HFD and resulting obesity enhanced fibrosis in *Pd*–KO mice.

The gonadal fat had similar, but less pronounced, changes compared to the inguinal fat (Supplemental Fig. S4b–d) with the exception that the inflammatory markers *Ifnγ, Tnf*, and *Il1β* were lower in *Pd*–KO mice on HFD compared to F/F controls (Supplemental Fig. S4d).

### 2.5 A new trajectory emerged after deletion of Mgp in Pdgfra-expressing cells

**Figure 6:**
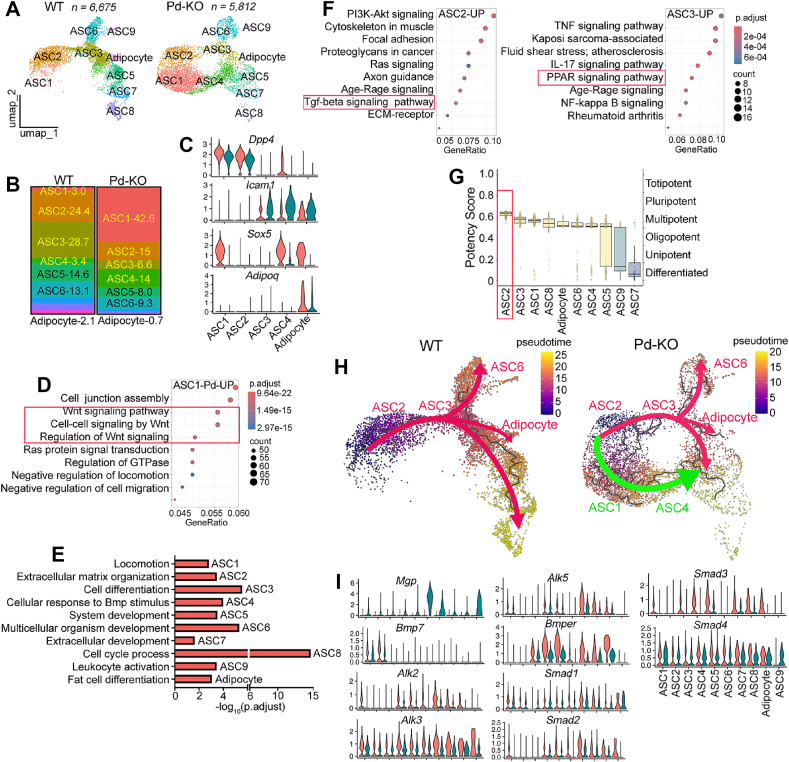
Pseudotime and CytoTRACE analyses identify a new cell trajectory after *Mgp* deletion in Pdgfrα-expressing cells and early adipocytes. A. Uniform Manifold Approximation and Projection (UMAP) plot of 12,487 cells of adipose progenitor cells (APCs) and early adipocytes from wild-type (WT) and *Pd*–KO mice. B. Boxplots showing the proportion of APCs and adipocytes from WT, and *Pd*–KO mice. C. VlnPlot of *Dpp4, Icam1, Sox5,* and *Adipoq* in clusters ASC1-4, and adipocytes from WT and *Pd*–KO adipose tissues. D. DotPlot of up-regulated KEGG pathway in the ASC1 cluster of the *Pd*–KO mice, as compared to the ASC2 cluster in WT mice. E. Gene Ontology Biological process analysis using the top50 genes in each cell type, as determined by g:Profiler. F. Comparison of the ASC2 and ASC3 clusters in WT adipose tissue; DotPlots of the up-regulated KEGG pathways in ASC2 (left) and in ASC3 (right). G. Potency scores for different cell clusters from the *Pd*–KO mice, as predicted by CytoTRACE2. The result suggests that the ASC2 cluster represents the earliest APCs. H. Trajectory analysis of the APCs (*Pdgfrα*+) and adipocyte clusters using Monocle3. UMAP visualization of APCs and adipocyte trajectories from *Pd*–KO and WT adipose tissue. I. VlnPlot of TGFβ pathway-related gene expression in WT and *Pd*–KO adipose tissues, including *Mgp, Bmp7, Alk2, Alk3, Alk5, Bmper, Smad1, Smad2, Smad3,* and *Smad4*.

Since deletion of *Mgp* in the *Pdgfra*-expressing progenitor cells affected cell differentiation, we examined the APC-populations. We used the same approach as above to perform scRNA-seq on inguinal fat pads from 11 *Pd*–KO mice (5 female, 6 male). The scRNA-seq results showed that the *Pd*–KO mice had similar cell categories (such as APCs, ECs and SMCs) as the *Mgp*-KO and wild-type mice. We captured 5,812 high quality progenitor cells (*Pdgfra*+) and early adipocytes (*Adipoq+*); re-clustering showed that they also separated into nine ASC clusters and one adipocyte cluster (Figure 6A).

The ASC1 and ASC2 clusters showed high expression of *Dpp4*, *Cd55*, and *Pi16*. The ASC1 cluster represented 42.6% of the APCs in *Pd*–KO mice versus only 3% in the control mice (Figure 6B). ASC1 was a subpopulation mainly associated with *Mgp* deletion that also had high expression of *Sox5* (Figure 6C). Analysis of the differentially expressed genes (DEGs) in the ASC1 cluster from the *Pd*–KO mice and in the ASC2 cluster from controls revealed enrichment of genes associated with the Wnt signaling pathway and its regulation in *Pd*–KO mice (Figure 6D).

The ASC3 and ASC4 clusters showed similar expression of *Icam1*, *Bmper*, *Cd36*, *Apoe*, and *Plin2*, but only the ASC4 cluster expressed *Dpp4*. Gene Ontology (GO) analysis of the top50 genes in the ASC4 cluster from the *Pd*–KO mice revealed enrichment of genes associated with the cellular response to BMP and regulation of supramolecular fiber organization (Figure 6E). KEGG pathway analysis of DEGs in the ASC2 and ASC3 clusters from 4-week-old wild-type mice scRNA-seq data found a strong enrichment of TGFβ pathway genes in ASC2, whereas the PPAR signaling pathway, essential for adipogenesis, was enriched in ASC3 (Figure 6F). There was only 6.6% ASC3 cells in the *Pd*–KO mice, compared to 28.7% in controls (Figure 6B). It suggested that deletion of *Mgp* in the *Pdgfrα*+ cells diverted these cells away from differentiation into preadipocytes, resulting in smaller inguinal fat pads in the *Pd*–KO mice.

The ASC1 and ASC4 clusters are rare cell populations in normal development, as shown by examining scRNA-seq data from wild-type mice of different ages including postnatal day 12 (p12, GSM3717977) [12], 4 weeks (GSM:8973244) and 10 weeks (10W, GSM3717978) [12] (Supplemental Fig. S5a–c). As the *Mgp* expression increased and peaked during this time period, the expression of TGFβ superfamily related genes such as *Bmp2*, *Bmp4*, and *Bmp7* gradually decreased (Supplemental Fig. S5d).

Separate Cytotrace2 analysis of *Pd*–KO and wild-type data found that the ASC2 cells have a high potency score, indicating that the ASC2 cluster represents earlier adipose progenitors (Figure 6G). It was therefore selected as the root for the Pseudotime trajectory analysis. The analysis revealed that under normal conditions, *Dpp4*+;*Sox5*-progenitor cells (ASC2) would differentiate to *Icam1*+;*Cd36*+ preadipocytes (ASC3) and later to adipocytes (Figure 6H).

Importantly, after *Mgp*-depletion, a new and predominant trajectory emerged in the *Pd–KO* data, where *Dpp4*+;*Sox5*-cells (ASC2) would differentiate to *Dpp4*+;*Sox5*+ cells (ASC1), and later transition to ASC4 (Figure 6H). The ASC4 cluster showed enrichment of genes related to the cellular response to BMP stimulus and regulation of supramolecular fiber organization (Figure 6C). The ASC4 also has *Dpp4*+ expression but less than in ASC1 and ASC2 (Figure 6C).

To further examine the *Pdgfra*+;*Dpp4*+ cells, we isolated these cells from inguinal fat from *Pd–*KO and control mice by FACS. The *Pd*–KO mice had a larger population of the *Pdgfra*+;*Dpp4*+ cells, which expressed higher levels of *Col1a1, Col3a1, Col6a1, and Pdgfra*, whereas *Adipoq* was decreased compared to controls (Supplemental Fig. S6a and b)*.* We induced adipogenesis in the wild-type *Pdgfra*^+^;*Dpp4*^+^ cells and treated with TGFβ1 (0–20 ng/ml). The results showed increased expression of *Col1a1* and *Tgfb1*, but reduced expression of *Adipoq,* and *Pdgfra* as the TGFβ1 concentration increased, resembling the *Pd*–KO cells (Supplemental Fig. S6c). Furthermore, the scRNA-seq result showed a strong enrichment of TGFβ-related components such as *Bmp7*, *Alk2*, *Alk3*, *Alk5*, *Bmper*, *Smad2* and *Smad3* in the *Pd*–KO fat pads compare to F/F controls (Figure 6I) suggesting activation of TGFβ signaling. Altogether, the data suggested that MGP has a coordinating role in BMP/TGFβ signaling, allowing BMP signaling to contribute to adipogenic differentiation in the APCs, while limiting TGFβ signaling that would accelerate fibrosis and suppress adipogenesis [32,34,41].

The remaining clusters were part of the normal trajectory and continued to be present in the trajectory after *Mgp*-deletion although in diminished amounts. The ASC6 cluster showed high expression of *F3/Cd142*, *Mgp*, *Gdf10*, *C2*, and *Vit* and corresponded to the previously described adipogenesis regulatory cells (Aregs, F3) [12]. The ASC5 and ASC7 clusters showed similarities in gene expression, including *Clo18a1*, *Sdc1*, and *Nxn*. However, the respective clusters also had distinct gene expression, such as *Cdh11*, *Fgf10* in the ASC5 cluster, and *Slit2*, *Frem1* in the ASC7 cluster (Figure 3G, Supplemental Fig. S5c).

### 2.6 ALK5 inhibition limits adipose fibrosis caused by Mgp deletion

Our scRNA-seq results suggested two possibilities of modulating the fibrogenic response. The first would be to suppress the TGFβ signaling, which is known to enhance fibrogenesis, and the second would be to inhibit the enzymatic activity of DPP4.

**Figure 7:**
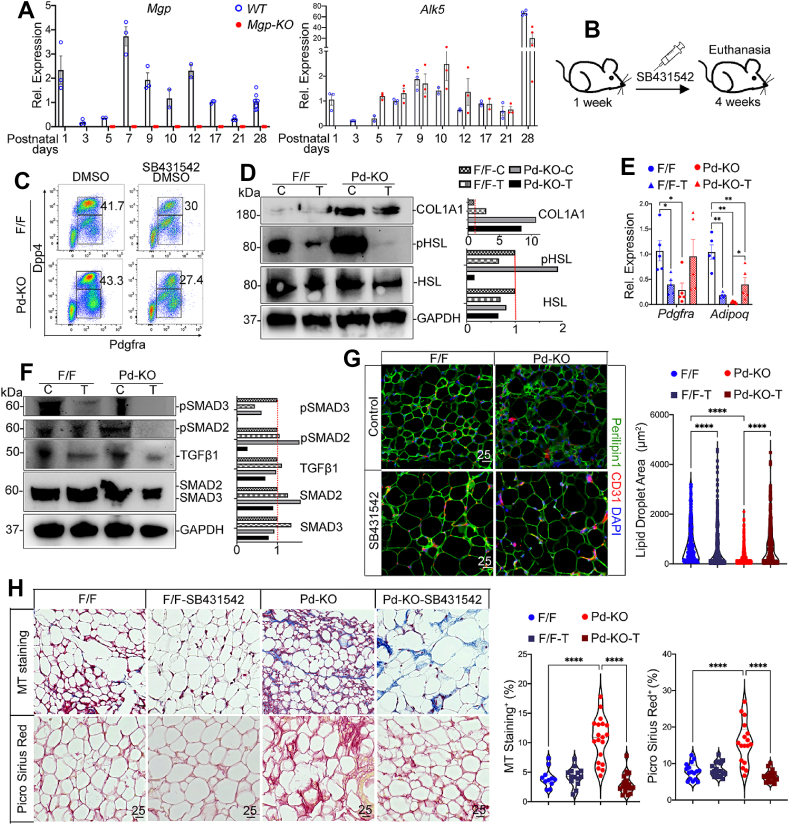
ALK5 inhibition limits adipose fibrosis caused by Pdgfrα Cre-mediated Mgp deletion. A. Time course expression of *Mgp* and *Alk5* in inguinal adipose tissue from wild-type (WT) and global *Mgp* knockout mice (*Mgp-*KO). *Mgp* expression peaked around postnatal day (P)7; *Alk5* expression peaked around P9-10. B. Schematic diagram of treatment with the ALK5 inhibitor SB431542 (10 mg/kg) in 10% DMSO or control 10% DMSO. *Mgp*^f/f^ (F/F) and *Mgp*^f/f^,*Pd*^Cre^ (*Pd*–KO) mice were injected daily from P7 to P28. The experiments in panels C-H were performed using inguinal adipose tissue from F/F and Pd–KO mice after SB431542 or control treatment. Each group has the same number of mice. C. FACS analysis of the stromal vascular fraction (SVF) for detection of early adipose progenitor cells (PDGFRα+; DPP4+), after removal of immune and endothelial cells, from F/F and *Pd*–KO mice (*n* = 4 mice per group). Percent (%) cells, representative of 3 experiments. D. Protein expression of hormone-sensitive lipase (HSL), phospho(p)-HSL, and COL1A1, as determined by immunoblotting with densitometry (protein from *n* = 5; representative of 3 experiments). E. Gene expression of *Pdgfrα,* and *Adipoq,* as determined by qPCR (*n* = 5). F. Protein expression of SMAD2, SMAD3, pSMAD2, pSMAD3, and TGFβ1 (*n* = 5), as determined by immunoblotting with densitometry (protein from *n* = 5; representative of 3 experiments). G. Immunofluorescence for Perilipin-1 (green) for lipid droplet (LD) size), and CD31 (red). DAPI (blue) was used to visualize nuclei. The LD size was quantified by ImageJ (*n* = 3). H. Masson’s trichrome (MT) and Picrosirius Red staining, quantified by ImageJ (*n* = 3). Data are shown as mean ± SEM; One way ANOVA, ∗*p* < 0.05, ∗∗*p* < 0.01, ∗∗∗*p* < 0.001, ∗∗∗∗*p* < 0.0001. Scale bars, 25 μm.

To better assess when to inhibit TGFβ signaling, we compared the expression time course of *Mgp* and the TGFβ receptor *Alk5* in inguinal fat tissue from *Mgp*-KO and wild-type mice. *Mgp* peaked on postnatal day (P)7 in wild-type, followed by a gradual decline to P21 (Figure 7A). *Alk5* expression increased from P3 and peaked on P9 in wild-type, and on P10 in *Mgp*-KO inguinal fat tissue (Figure 7A). Thus, *Mgp* peaked before *Alk5*, suggesting that inhibition of TGFβ signaling should start around P7.

To determine the effect of ALK5 inhibition *in vivo*, we treated *Pd–*KO and F/F control mice with the ALK5-inhibitor SB431542 or DMSO control through daily I.P. injections for 3 weeks, starting at one week of age (Figure 7B). The treatments were stopped one day before euthanasia. Without treatment, the *Pd*–KO inguinal fat had a larger population of PDGFRα+; DPP4+ cells than controls, which is consistent with our scRNA-seq results. After SB431542 treatment, the PDGFRα+; DPP4+ cell population decreased significantly in both *Pd*–KO and F/F mice, from 43.3% to 27.4 % and 41.7%–30%, respectively (Figure 7C). Prior to treatment, COL1A1 and the phosphorylated form of hormone-sensitive lipase (pHSL), a crucial enzyme in lipolysis, were elevated in the *Pd*–KO mice compared to controls, as determined by immunoblotting (Figure 7D). However, SB431542 reduced COLA1A, HSL and pHSL in both *Pd*–KO and F/F control mice (Figure 7D). Furthermore, SB431542 enhanced the expression of *Pdgfra* and *Adipoq* in the *Pd*–KO mice, as determined by qPCR (Figure 7E). We also determined the effect of SB431542 on TGFβ canonical SMAD2/3 activation in inguinal fat tissue. The results showed suppression of pSMAD2 and pSMAD3 in both mice after treatment, as determined by immunoblotting (Figure 7F). The level of TGFβ1 was also suppressed. The lipid accumulation, as visualized by Perilipin-1 staining, was initially smaller in the *Pd*–KO mice than the controls but increased in size after treatment (Figure 7G). MT staining, Picrosirius Red-stained collagens, and immunofluorescence for fibronectin all decreased in the *Pd*–KO mice after the SB431542 treatment compared to controls (Figure 7H, Supplemental Fig. S7). Together, the results showed that suppression of TGFβ signaling limited fibrosis caused by *Pdgfra*-targeted *Mgp* deletion.

### 2.7 MGP modulated TGFβ signaling in mesenchymal stem cells *in vitro*, affecting adipogenesis and fibrosis

**Figure 8:**
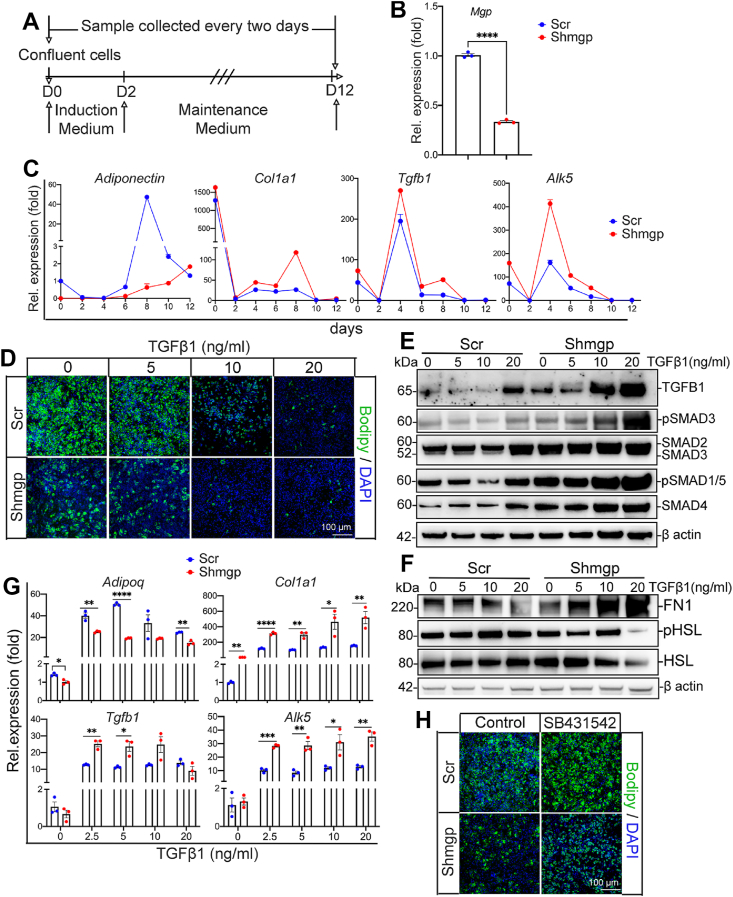
MGP modulates the TGFβ pathway in C3H10T1/2 mesenchymal progenitor cells affecting adipogenesis and fibrosis *in vitro*. C3H10T1/2 cells transfected with lentiviral vectors were used to generate stable cell lines labeled *Shmgp* for *Mgp* knockdown or scrambled (*Scr*) control. The experiments were replicated 3 times. A. Schematic diagram of adipogenic induction, maintenance and sample collection in C3H10T1/2 cells. B. Expression of *Mgp* in *Shmgp* or *Scr* transfected cells. *Shmgp* suppressed expression of *Mgp* about 70% compared to *Scr* control on day 0. C. Time course expression of *Adipoq, Col1a1, Tgfβ1,* and *Alk5* in *Shmgp* or *Scr* transfected cells, as determined by qPCR. D. Bodipy staining of *Shmgp* or *Scr* transfected cells after 12 days of treatment with different concentration of TGFβ1 (0–20 ng/ml). E, F. Expression of (E) SMAD4, SMAD2/3, pSMAD3, TGFβ1, and pSMAD1/5, and (F) Fibronectin (FN1), hormone-sensitive lipase (HSL), phospho(p)-HSL in *Shmgp* or *Scr* transfected cells, after 12 days of treatment with different concentrations of TGFβ1 (0–20 ng/ml), as determined by immunoblotting with densitometry (representative of 3 experiments). G. Expression of *Adipoq, Col1a1, Tgfb1,* and *Alk5* after 12 days of treatment with different concentration of TGFβ1 (0–20 ng/ml), as determined by qPCR. H. Bodipy staining of *Shmgp* or *Scr* transfected cells after 12 days of treatment with 5 μM SB431542. Data are shown as mean ± SEM; One way ANOVA, ∗*p* < 0.05, ∗∗*p* < 0.01, ∗∗∗*p* < 0.001, ∗∗∗∗*p* < 0.0001.

To further examine whether MGP balanced adipogenesis and fibrosis in progenitor cells through the TGFβ pathway, we used the mesenchymal stem cells C3H10T1/2. We suppressed *Mgp* with lentiviral vectors containing *Shmgp* or scrambled *Scr* control, achieving a knockdown efficiency of over 70% with *Shmgp* (Figure 8A–B). In response to the *Mgp* reduction, the expression of the *Col1a1*, *Alk5*, and *Tgfb1* increased compared to controls, as shown by qPCR time course expression, whereas *Adipoq* expression decreased (Figure 8C). It suggested that MGP suppressed adipogenesis while enhancing fibrosis in the C3H10T1/2 cells.

We then treated the *Shmgp*- and *Scr*-transfected cells with TGFβ1 (0–20 ng/ml) for 12 days and stained with Bodipy and DAPI to visualize lipids and nuclei, respectively. After 12 days of TGFβ1 treatment, the results showed a reduction in lipid accumulation as the TGFβ1 concentration increased in both *Shmgp* and *Scr* cells (Figure 8D). The same TGFβ1 dose resulted in less lipid accumulation in the Shmgp cells. In addition, the protein levels of TGFβ1, SMAD2, SMAD3, SMAD4, pSMAD3 and pSMAD1/5 increased with TGFβ1 dose, and were higher in the *Shmgp* cells under the same dose (Figure 8E). The expression of *Col1a1*, *Tgfb1*, and *Alk5* as well as the protein level of fibronectin increased, whereas expression of *Adipoq* and the protein levels of HSL and pHSL were reduced in *Shmgp* cells compared to *Scr* controls (Figure 8F,G). Furthermore, treatment of the *Shmgp* cells with SB431542 (5 μM) for 12 days showed an increase in lipid accumulation, as shown by Bodipy staining (Figure 8H). Together, the results suggested that loss of *Mgp* activated TGFβ signaling and diverted progenitor cells toward fibrogenesis.

### 2.8 DPP4 inhibition limited adipose fibrosis caused by Mgp deletion

ScRNA-seq data showed that *Pdgfrα*+;*Dpp4*+ double positive cells (ASC1 + ASC2 + ASC4) constituted 57.6% of APCs in the *Pd*–KO inguinal fat deposits, compared to only 27.4% in controls (Figure 6B). The scRNA-seq showed that the *Dpp4*+ cells were highly enriched in components of the TGFβ pathway, also noted in previous studies by Merrick et al. [12]. We speculated that a reduction in DPP4 enzymatic activity would reduce the fibrosis caused by *Mgp* deletion.

**Figure 9:**
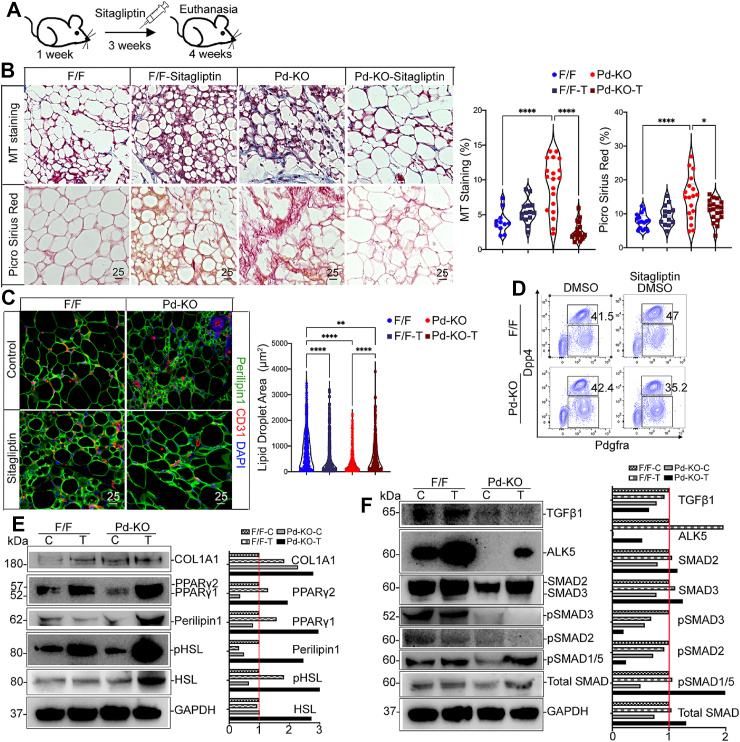
DPP4 inhibition limits adipose fibrosis caused by *Pdgfrα* Cre-mediated Mgp deletion. A. Schematic diagram of treatment for mice with sitagliptin (10 mg/kg) in 5% DMSO or control 5% DMSO. *Mgp*^f/f^ (F/F) and *Mgp*^f/f^,*Pd*^Cre^ (*Pd*–KO) mice were injected daily from P7 to P28. The experiments in panels b-f were performed using inguinal adipose tissue from F/F and Pd–KO mice after sitagliptin or control treatment. Each group has the same number of mice. B. Masson’s trichrome (MT) and Picrosirius Red staining, quantified by ImageJ (*n* = 3). C. Immunofluorescence for Perilipin-1 (green) and CD31 (red) in inguinal adipose tissue from F/F and *Pd*–KO mice at 4 weeks. DAPI (blue) was used to visualize nuclei. Bars, 25 μm. The LD size was quantified by ImageJ (*n* = 3). D. FACS analysis of the stromal vascular fraction (SVF) for detection of early adipose progenitor cells (PDGFRα+; DPP4+), after removal of immune and endothelial cells in inguinal adipose tissue from F/F and *Pd*–KO mice (*n* = 4 mice per group). Percent (%) cells is representative of 3 experiments. E. Expression of Pparγ1, Pparγ1, Perilipin-1, hormone-sensitive lipase (HSL), phospho(p)-HSL, and COL1A1, as determined by immunoblotting with densitometry (protein from *n* = 5; representative of 3 experiments). F. Expression of SMAD2, SMAD3, pSMAD3, pSMAD2, pSMAD1/5, TGFβ1, ALK5, and total SMAD, as determined by immunoblotting with densitometry (protein from *n* = 5; representative of 3 experiments). Data are shown as mean ± SEM; One way ANOVA, ∗*p* < 0.05, ∗∗*p* < 0.01, ∗∗∗∗*p* < 0.0001.

Sitagliptin is a reversible DPP4 inhibitor often used in the treatment of type 2 diabetes. To determine the effect of modulating the DPP4 activity in the *Pd*–KO mice *in vivo*, we treated the *Pd*–KO mice and F/F controls with injections of sitagliptin or DMSO control daily for 3 weeks, starting at one week of age (Figure 9A). After sitagliptin treatment, MT staining, Picrosirius Red-stained collagens, and immunofluorescence for fibronectin decreased in the *Pd*–KO mice, (Figure 9B, Supplemental Fig. S8). The lipid droplet size in the *Pd*–KO mice was slightly increased after treatment with sitagliptin, but its size was still reduced compared with treated and untreated control mice (Figure 9C). FACS analysis showed that the PDGFRα+; DPP4+ cell population was moderately reduced in *Pd*–KO mice from 42.4% before treatment to 35.2% after treatment (Figure 9D). Perilipin-1, the adipogenic-related transcription factors PPARγ1 and PPARγ2, HSL and pHSL were enhanced by sitagliptin in both mice, with the largest increase in the *Pd*–KO mice, as determined by immunoblotting (Figure 9E). However, COL1A1 was not reduced to the same extent as fibronectin (Figure 9E), suggesting that sitagliptin may have variable effects on ECM components. Finally, we examined the effect of sitagliptin on TGFβ signaling. The results showed that sitagliptin strongly enhanced ALK5 levels in both *Pd*–KO and control mice, whereas the levels of TGFβ1 and pSMAD3 decreased (Figure 9F), supporting an overall suppression of the TGFβ signaling. In addition, BMP-activation was suggested by the increased pSMAD1/5 levels in the *Pd–KO* mice after treatment (Figure 9F). The results suggested that the DPP4 inhibitor sitagliptin may limit the fibrosis caused by the lack of MGP through modulation of the TGFβ and BMP pathways.

## 3 Discussion

Improved control of adipose fibrosis may have profound benefits in the treatment of chronic disease such as type 2 diabetes, metabolic dysfunction and cardiovascular disease. In our study, we found that global *Mgp* deletion promoted adipose fibrosis while inhibiting white adipogenesis. Furthermore, we found that targeting of the *Mgp* deletion to *Pdgfra*-expressing cells was sufficient to generate excess fibrosis. ScRNA-seq identified a population of *Pdgfrα*+;*Dpp4*+ cells closely linked to both adipogenic and fibrogenic differentiation. Inhibition of ALK5 or DPP4 with SB431542 or sitagliptin, respectively, in *Pd*–KO mice reduced TGFβ signaling, the size of the PDGFR*α*+; DPP4+ cell population and ultimately adipose fibrosis.

Merrick et al. [12] previously reported that the DPP4+ cell population transitions to ICAM1+ preadipocytes prior to differentiating into adipocytes. It is also known that the DPP4+ cell population as well as the DPP4 activity increase during the development of fibrosis in heart, lungs, liver and kidneys[[14], [15], [16], [17], [18], [19]]. DPP4 was identified as a marker for activated fibroblasts in systemic sclerosis [13], and inhibition of DPP4 limits ECM deposition and fibrosis in heart, lungs, liver and kidneys [[14], [15], [16], [17], [18], [19]], suggesting an active contribution to fibrosis. Our results suggest that both DPP4 and MGP affects the decision to undergo adipogenic differentiation versus fibroblast activation. Our scRNA-seq results point to involvement of three clusters of *Pdgfra*+;*Dpp4*+ cells, the ASC1, ASC2 and ASC4 clusters. The ASC1 and ASC4 clusters were highly responsive to *Mgp* deletion.

The ASC1 cluster is novel and was found predominantly in the MGP-deficient mice. It represents only 3% of the wild-type cells compared to 42.6% of the *Pd*–KO cells, suggesting that its emergence is part of an MGP-deficient phenotype, even though the *Mgp* expression in the cells themselves is relatively low. The ASC2 cluster, on the other hand, is present in both wild-type and MGP-deficient mice. These cells show high expression of *Dpp4*+, *Pi16*+, *Cd55*+, which is similar to both the ASC2 cells (*Dpp4*+, *Pi16*+) reported by Burl et al. [42] and the group 1 cells reported by Merrick et al. [12]. The ASC2 cells are present during several stages of normal adipose development, including postnatal day 12, and 4 and 10 weeks in the age-related analysis (Supplemental Fig. S5a–c). Our results showed that the ASC2 population represented 24.4% of wild-type cells, compared to 15% of *Pd*–KO cells (Figure 6B). The ASC3 cluster is similar to the p2 cluster reported by Schwalie et al. [43], and the group 2 cells reported by Merrick et al. [12]. The ASC3 cluster is a preadipocyte population that represents 28.7% of wild-type cells but only 6.6% of *Pd*–KO cells. It is consistent with an effect of MGP on the transition of DPP4+ cells in the ASC2 cluster to ICAM1+ preadipocytes in the ASC3 cluster.

In attempt to understand how MGP affects the transition of DPP4+ cells to ICAM1+ preadipocytes, we examined the DEGs and found enrichment of the TGFβ superfamily pathways in the ASC2 cluster. The TGFβ-receptors *Alk2*, *Alk3*, and *Alk5,* as well as the downstream transcription factors *Smad2* and *Smad3* were increased in the *Pd*–KO cells (Figure 6I). We predicted that loss of *Mgp* would activate the TGFβ pathway and divert PDGFRα+; DPP4+ cells to fibrogenic differentiation. Indeed, the ALK5 inhibitor SB431542 reduced the PDGFRα+; DPP4+ cell population and largely normalized the adipocytes in the *Pd*–KO mice (Figure 7C,G). It suggests that the reduction in TGFβ1, pSMAD2 and pSMAD3 (Figure 7H) rescued the fibrosis resulting from *Mgp* deletion. We also observed increased levels of BMP-activation, noted in previous work to be upstream of TGFβ activity [20,21].

The adipose fibrosis was accompanied by an increase in the size of the PDGFRα+; DPP4+ cell population. Both SB431542 and sitagliptin were able to suppress this cell population, which reduced the fibrosis and supported the adipocytes in the *Pd*–KO tissues. However, the reduction in PDGFRα+; DPP4+ cells was only moderate after sitagliptin treatment, from 42.4% to 35.2% (Figure 9D). Nonetheless, sitagliptin rescued the fibrosis phenotype in the *Pd*–KO mice. Although sitagliptin increased ALK5 in *Pd*–KO tissues, it was without pSMAD3 activation (Figure 9F), suggesting that ALK5 signaling was not activated through the SMAD pathway (Figure 9F). We speculate that sitagliptin may have selectively stimulated a slightly later transition already under way toward PDGFRα+; ICAM1+ cells (ASC3, preadipocytes) rather than ASC1 and ASC4.

It is known that BMP4 plays an important role in the commitment of mesenchymal stem cells to preadipocytes and differentiation to adipocytes [29,44,45]. Analysis of scRNA-seq results from different ages found that expression of *Bmp4* in early wild-type APCs diminished as *Mgp* increased (Supplemental Fig. S5d). Normal BMP and TGFβ regulation support APCs and early adipocytes during development and maintain a source of preadipocytes in adults. Without normal levels of MGP, excess BMP and TGFβ activation would be expected to persist beyond the developmental time frame and disrupt differentiation. The excess TGFβ1 would affect APCs with high *Alk5* expression and promote fibrogenesis.

Both adipose expression and serum levels of BMP4 are increased in obesity [46,47]. BMP4 may be a mediator of inflammation [48] but has also been reported as protective against inflammation [49]. BMP4 is also important for endothelial differentiation and tissue vascularization [50], which is consistent with the increase in BMP activation and vascular density as MGP is depleted (Supplemental Fig. S3b–c). Ultimately, the effect of BMP4 may depend on when it is active in adipose tissue. BMP4 may be anti-inflammatory in APCs during development, but serve as an inflammatory mediator and pro-fibrotic factor in diseased tissues.

We preferred the *Pdgfrα-Cre* transgenic mice to the *Dpp4-Cre* transgenics, since expression of *Mgp* and *Pdgfrα* overlapped in the APCs and resulted in targeted *Mgp* depletion. The lack of reliable surface markers is a limitation in this and other studies. Such markers would help isolate and characterize the different ASC clusters for use *in vitro* studies. Furthermore, there is no certain way to reliably perform lineage tracing in the different clusters.

The role of *Mgp* in liver fibrosis was not addressed here but has been previously addressed in two models of diet-induced non-alcoholic steatohepatitis (NASH) [51]. *Mgp* was primarily expressed in hepatic stellate cells and dendritic cells in mice, and heterozygosity for global *Mgp*-deletion had a protective effect on hepatic fibrosis. Additional studies will be required to clarify potential links between the effects of MGP on adipose and hepatic fibrosis.

Other pathways of potential interest for future studies include the Wnt pathway, which interacts with DPP4 and TGFβ signaling and is known to inhibit adipogenesis [52,53]. DEG analysis found enrichment of genes belonging to the Wnt pathway in the ASC1 cluster derived from the *Pd*–KO inguinal fat (Figure 6D). We also found enhanced *Zpf423* expression in the ASC1, ASC4, and ASC5 clusters and early adipocytes from the *Pd*–KO mice (Supplemental Fig. S9a). Zfp423 is important for terminal differentiation of subcutaneous white adipocytes, and mice deficient in *Zpf423* show abnormal expansion of the inguinal fat [54] which did not occur in our studies. Finally, we noted that deletion of *Mgp* increased several collagens (*Col1a1, Col3a1, Col6a1)* in the PDGFRα+; DPP4+ cells (Supplemental Fig. S6b). *Col6a1* may be of special interest since it has been shown to regulate adipogenic and lipolytic phenotypes [55,56]. There was also variability in the expression of the subtypes *Col6a1*, *Col6a2* or *Col6a3* in the different ASC clusters in *Pd*–KO mice and controls (Supplemental Fig. S9b).

In summary, MGP is essential in regulating the fate of *Pdgfra*+;*Dpp4*+ cells by balancing adipose BMP and TGFβ signaling. Deletion of *Mgp* causes failure in APC regulation and diverts the *Pdgfra*+;*Dpp4*+ cells towards fibroblast activation and fibrosis (Supplemental Fig. S10 for schematic model). Reduction of TGFβ signaling, DPP4 activity or the PDGFRα+; DPP4+ cell population rescued the WAT from unwanted fibrosis.

## 4 Material and methods

*Key Resources* – see Supplemental Table 1.

### 4.1 Contacts for reagent and resource sharing

Requests for further information, resources, and reagents should be directed to the corresponding authors Li Zhang (LiZ@mednet.ucla.edu) and Kristina I. Boström (kbostrom@mednet.ucla.edu). Sharing of primary samples is based on availability and may be subject to Material Transfer Agreements (MTAs) and will require appropriate research ethics board certifications.

### 4.2 Animals

*Mgp*^*+/−*^ mice (B6.129S7-Mgptm1Kry/KbosJ, Stock No: 023811), *Pdgfra*^*cre*^ (C57BL/6-Tg (Pdgfra-cre)1Clc/J Stock No: 013148) were obtained from the Jackson Laboratory. *Mgp*^*flox/flox*^ (F/F) mice were generated in our laboratory as previously described [57]. *Pd*–KO mice were generated by crossbreeding of *Mgp*^*flox/flox*^ and *Pdgfra*^*cre*^ mice. All mice were exposed to a standard 12h:12h light/dark cycle and fed a standard chow diet (Diet 8604; Harlan Teklad Laboratory). The mice were weaned 21 days after birth, and male and female mice were separated. Since male and female phenotypes were similar, mixed gender groups were analyzed together. The studies were reviewed and approved by the Institutional Review Board and conducted in accordance with the animal care guidelines set by the University of California, Los Angeles. The investigation also conformed to the standards of National Research Council (NRC), *Guide of the Care and Use of Laboratory Animals, Eight Edition* (Washington, DC; The National Academies Press, 2011).

### 4.3 High fat feeding of mice

Each gender was divided into two groups with at least 5 mice per group. Both groups were initially fed a regular chow diet (CD; Teklad Laboratory: 8604) until 8 weeks of age. At 8 weeks of age, one group of F/F and *Pd–KO* mice was placed on a high-fat diet (HFD; 45 kcal% Fat, Research Diets: D12451) for 20 weeks to induce obesity, while the other same group was continued on the chow diet. Body weight was determined by NMR every two weeks. All mice were fasted for 4 h before euthanasia with isoflurane.

### 4.4 Mouse body composition determination

Mouse body weight, body fat and lean muscle mass were determined by a Bruker Optics Minispec NMR analyzer machine (Billerica, MA) according to the manufacturer’s recommendations.

### 4.5 Glucose tolerance test (GTT)

Mice were transferred into new cages with fresh bedding, and fasted for 14 h before the GTT test. The mice were fed 2.5 g/kg glucose by oral gavage. Blood samples were collected from the tail vein at 0, 15, 30, 60, 90 and 120 min post gavage. Blood glucose was measured using a glucometer and glucose test strips (AlphaTRAK 2 test strips).

### 4.6 Insulin tolerance test (ITT)

Mice were fasted for 4 h before insulin injection (0.75 U/kg) through intraperitoneal injection. Blood samples were collected from the tail at 0, 15, 30, 45, 60 and 120 min post injection, and blood glucose was measured using a glucometer and glucose test strips (AlphaTRAK 2 test strips).

### 4.7 Lipid analysis

Serum L-Type triglyceride M (L-TG) and Non-esterified fatty acids (NEFA) were measured using a colorimetric biochemical assay (Wako Chemicals USA) according to the manufacturer’s protocol. The processed samples were measured OD 600 nm for L-TG and OD 550 nm for NEFA using a SpectraMax 384 Plus Microplate Spectrophotometer (Molecular Devices).

### 4.8 Mouse treatments

*Mgp*^*flox/flox*^; *Pdgfrα*^*cre*^ (*Pd*–KO) and *Mgp*^*fl*^^*ox*^^*/fl*^^*ox*^ (F/F) control mice were equally and randomly assigned to treatment and control groups. Intraperitoneal (IP) injections were performed once daily, 5 days per week, with no treatment on weekends. The treatments were initiated at one week of age and continued until 4 weeks of age. The treatment was stopped 24 h before sacrificing the mice. For SB431542 treatment, one group each of *Pd*–KO and F/F mice were injected with 10 mg/kg SB431542 in 10% dimethyl sulfoxide (DMSO) solution, whereas the control groups were injected with 10% DMSO control. For sitagliptin treatment, one group each of *Pd*–KO and F/F mice were injected with 10 mg/kg sitagliptin in 5% DMSO, whereas the control groups were injected 5% DMSO control.

### 4.9 Transfection of C3H10T1/2 cells with shRNA lentiviral particles

MGP shRNA (*Shmgp*) and control shRNA (*Scr*) (Santa Cruz Biotechnology, #sc-44627-v and #sc-108080, respectively) were transfected into C3H10T1/2 cells (ATCC, #CCL-226) using 5 μg/ml of the Polybrene Transfection Reagent (Sigma–Aldrich, #TR-1003-G) according to the manufacturer’s instructions. Stable clones were selected using puromycin (Gibco™, #A1113803) for one week. RT-qPCR was performed to assess the efficiency of the *Mgp* suppression.

### 4.10 Adipogenic cell culture

The C3H10T1/2 cells were selected for these experiments since they may represent mesenchymal stem cells at stages prior to pre-adipocytes. C3H10T1/2 cells transfected with lentiviruses MGP shRNA (*Shmgp*), and control shRNA (*Scr*) were cultured in Dulbecco’s Modified Eagle Medium (DMEM) supplemented with 20% fetal bovine serum (FBS), 100 U/mL penicillin, and 100 mg/mL streptomycin. The protocol for adipose differentiation was carried out as previously described and started when the cells were at >95% confluency [27,58]. The near-confluent cells were treated with induction medium, which contained growth media (DMEM with 10% FBS) supplemented with 5 mg/mL insulin, 125 mM indomethacin, 1 nM 3,3′,5-Triiodo-l-thyronine (T3), 2 mg/mL dexamethasone, and 0.5 mM 3-Isobutyl-1-methylxanthine, for 48 h (day 0 to day 2). After 48 h, the cells were treated with maintenance medium containing the growth media supplemented with 5 mg/mL insulin, 1 nM T3. The medium was changed every 2 days. Samples were collected every 2 days until day 12.

*Shmgp* and *Scr* transfected C3H10T1/2 cells were treated with 0, 5, 10, and 20 ng/ml of mouse recombinant TGFβ1 (R&D Systems, #7666-MB/CF) in the induction medium for 48 h (day 0 to day 2), or control medium without TGFβ1. Samples for RT-qPCR and immunoblotting were collected on day 12. *Shmgp* and *Scr* transfected C3H10T1/2 cells were also treated with 5 μM SB431542 and staining with Bodipy™ 493/503 (below) was performed on day 12.

### 4.11 Isolation of stromal vascular fraction (SVF) cells

SVF cells from subcutaneous (inguinal) white adipose tissue were isolated, using a modification of a previously described methodology [27]. Briefly, the inguinal fat was dissected, minced, and digested in 0.1% (w/v) collagenase II solution (Sigma, #C6885) at 37 °C for 30 min with gentle agitation. DMEM supplemented with 20% FBS was used to stop the digestion. The digested adipose tissue was filtered through a 100 μm-cell strainer and centrifuged at 500 rpm for 5 min. The pelleted layer included the SVF cells, and blood cells, which were disrupted using Erythrocyte lysis buffer. The remaining SVF cells were washed twice with FACS buffer (5 mm ethylenediaminetetraacetic acid (EDTA) and 2% FBS in Dulbecco’s Phosphate-Buffered Saline (DPBS), pH 7.4).

### 4.12 Fluorescence-activated cell sorting (FACS)

FACS was performed as previously described [59]. See Supplemental Table 1 for antibody information. SVF cells from inguinal adipose tissue were collected and resuspended in FACS buffer (5 mm EDTA and 2% BSA in DPBS, pH 7.4) for staining with the following antibodies for 1 h at 4 °C in the dark: CD31-PE (1:200), CD45-APC (1:100), dipeptidyl peptidase-4 (DPP4)/CD26 Alexa Fluor 594 (1:100), and platelet-derived growth factor receptor α (PDGFRα)-BV786 (1:100). 4',6-Diamidino-2-phenylindole (DAPI, 1:5,000), which was used to identify dead cells, was added during the last 10 min. The SVF cells were washed three times using the FACS buffer to remove unbound antibodies. Flow cytometer gates were set using unstained SVF cells as controls. Cells were gated by forward scatter versus side scatter to eliminate debris. DAPI-negative live cells were used for continued analysis. A minimum of 10,000 live cells were counted for each analysis. All FACS experiments were completed on an LSR II Flow Cytometer (BD Biosciences). The FACS results were analyzed and presented using FlowJoTMv10.7 software.

### 4.13 Immunoblotting

After euthanasia, the mouse inguinal adipose tissue was immediately dissected, frozen directly in liquid nitrogen, and stored at −80 °C until use. Approximately 100 mg adipose tissue from 4-week-old mice was homogenized in 500 μl RIPA buffer with protease inhibitors (Roche, 1:100) and phosphatase inhibitors (Roche, 1:100). Immunoblotting was performed as previously described. See Supplemental Table 1 for antibody information. The following primary antibodies from Cell Signaling Technology (CST) were diluted 1:1,000: peroxisome proliferator activated receptor gamma (PPARγ), Perilipin-1, hormone-sensitive lipase (HSL), phospho(p)-HSL, TGFβ1, SMAD2/3, p-SMAD3, p-SMAD1/5, and SMAD4. Primary antibodies against glyceraldehyde-3-phosphate dehydrogenase (GAPDH) from CST were diluted 1:5,000. Primary antibodies against Fibronectin (Sigma–Aldrich) and Collagen1A1 (Abcam) were diluted 1:2,000. The following day, the nitrocellulose membranes were washed with Tris-Buffered Saline with Tween (TBST) 3 times, 10 min each time, and incubated with horseradish peroxidase (HRP)-conjugated secondary antibodies for 1 h at room temperature. The nitrocellulose membranes were then washed 3 times in TBST, 10 min each time, and exposed in the ChemiDoc™ MP Gel Imaging System (Bio-Rad). The protein bands were quantified by densitometry using ImageJ Software.

### 4.14 Immunofluorescence

Immediately after euthanasia using isoflurane, the mice were perfused with 25 ml of 1x phosphate-buffered saline (PBS) per mouse, and the inguinal adipose tissue was dissected and fixed in 4% paraformaldehyde overnight. After washing twice with fresh water, the tissue was dehydrated and embedded in paraffin. Sections (5 μm) were cut from the paraffin blocks, and subsequently dewaxed, rehydrated and antigens were retrieved using unmasking solution. Sections were then incubated with primary antibodies. See Supplemental Table 1 for antibody information. The following antibodies and dilutions were used: Perilipin-1 (CST, 1:500), PDGFRα (R&D Systems, 1:200), CD31 (R&D Systems, 1:200), Fibronectin (Sigma–Aldrich, 1:200), MGP (Abcam, 1:2,000), and DPP4 (Abcam, 1:200). Alexa Fluor–conjugated secondary antibodies (1:500, Invitrogen) were applied and the sections were co-stained with DAPI (Sigma–Aldrich, 1:5,000). Staining without antibodies and with non-specific lgG primary antibodies served as controls. Image acquisition was performed with an inverted Nikon Eclipse Ti–S microscope (Nikon Corporation, Tokyo, Japan) or performed as Z-stacks using a KEYENCE BZ-X810 inverted fluorescence phase contrast microscope (Itasca, IL, USA).

The immunostaining was analyzed using ImageJ software. To determine the size of the lipid droplets, sections were stained for Perilipin-1, which outlined the lipid accumulation in the adipocytes. The image was adjusted to ensure that all adipocytes of interest were visualized. The lipid areas were then selected, measured (μm^2^) and analyzed. The results were shown as Vlnplot.

### 4.15 Bodipy™ staining

After induction of *Shmgp*- and *Scr*-transfected cells to mature adipocytes, the cells were washed gently twice with PBS, fixed in 4% paraformaldehyde, and stained with Bodipy™ 493/503 (Thermo Fisher Scientific, D3922) and DAPI as previously described [24].

### 4.16 Total collagen assay

Adipose tissue samples of the same weight and from corresponding tissue locations were processed and measured using the Total Collagen Assay Kit (Abcam, #ab222942) according to the manufacturer’s protocol. The processed samples were measured at OD 560 nm using a SpectraMax 384 Plus Microplate Spectrophotometer.

### 4.17 Masson’s trichrome (MT) and Picrosirius Red histochemical staining

Sections were deparaffinized, rehydrated and stained with Picrosirius Red Stain Kit (Abcam, ab150681) and Trichrome Stain Kit (Connective Tissue Stain, Abcam, ab150686) following the manufacturer’s protocols. After washing, the sections were dehydrated in fresh absolute ethanol and cleared in xylene, then mounted with resinous mounting medium.

### 4.18 Quantitative reverse transcription polymerase chain reaction (RT-qPCR)

Total RNA was extracted from cells or inguinal adipose tissue using RNeasy mini kits (Qiagen) and RNeasy lipid tissue Mini Kits. CDNA was obtained utilizing high-capacity cDNA reverse transcription kits (Thermo Fisher Scientific) according to the manufacturer’s instructions. RT-qPCR was measured on a 7500 Fast Real-Time PCR System (Applied Biosystems) with TaqMan Universal PCR Master Mix (Thermo Fisher Scientific). Cycle conditions included one cycle at 50 °C for 2 min, one cycle at 95 °C for 10 min, and then 40 cycles at 95 °C for 15 s and 60 °C for 1 min. Threshold cycles of specific cDNAs were normalized to the housekeeping gene *Gapdh* and translated to relative values. See Supplemental Table 2 for primers used in qPCR. Primers and probes for the mouse genes matrix Gla protein (*Mgp*), collagen 1a1 (*Col1a1*), adiponectin (*Adipoq*), fibronectin 1 (*Fn1*), *Tgfb1*, activin receptor-like kinase 5 (*Alk5*), *Pdgfra* were purchased from Thermo Fisher as part of Taqman® Gene Expression Assays.

### 4.19 Library preparation, sequencing and alignment

Single-cell RNA sequencing (scRNA-seq) cDNA libraries were generated with the Chromium Single Cell platform and 3′ v3 Reagent Kit based on manufacturer’s protocol (10x Genomics). The library was sequenced using Illumina NovaSeq SP platform to obtain 50-bp paired-end reads with a depth around 300 million reads per library. Cell Ranger (v6.1.1) was used with default parameters to align reads to the mouse genome (mm10).

### 4.20 Cell clustering and cell-type annotation

The R package Seurat (v.5.0.3) [60] was used to cluster the cells. Low-quality cells with <500 transcripts detected or >10% mitochondrial gene expression were first filtered out. We used LogNormalize with default scale factor 10,000 to normalize the feature expression for each cell divided by the total gene counts for the cell. The FindVariableFeatures function was used to select variable genes with default parameters. Later the dataset was scaled and the counts centered using the ScaleData function. Principal component analysis (PCA) was performed on the variable genes, and 30 principal components were used for cell clustering (resolution = 0.5) and Uniform manifold approximation and projection (UMAP) non-linear dimensionality reduction was used. Harmony was used for data integration [61]. The cluster markers were found using the FindAllMarkers function, and cell types were manually annotated based on the cluster markers. The sample composition was calculated relying on the number of cells for each cell type from each sample. This number was then divided by the total number of cells for each sample and scaled to 100% for each cell type [35].

### 4.21 Re-analysis of published data sets and 4 weeks wild-type scRNA-seq data

To compare our 4-week wild-type (WT) dataset with already published CD45 negative SVF cells from inguinal adipose tissue, we re-analyzed SVF cells from postnatal day 12 (GSM3717977), and 10 weeks (GSM3717978) [12] and our data from 4 weeks. The analysis followed our process (above) for cell clustering and cell-type annotation. The cells were annotated using our specifications.

### 4.22 Data analysis on Pdfgra-expressing cells

Cells with *Pdgfrα* expression level >0.1 were re-analyzed as described above with data normalization and clustering. To determine the composition of WT, *Mgp-KO* and *Pd–KO*, the cell numbers for each cluster of single cell data from WT, *Mgp-KO*, *Pd–KO* mice were counted, and percentages were calculated relative to total cell numbers.

### 4.23 Pathway analysis

We used Seurat function FindAllMarkers to identify differentially expressed genes (DEGs) in each cell cluster. The top50 or top20 genes in each cell cluster were put into g:Profiler [62] (https://biit.cs.ut.ee/gprofiler/gost) for Gene Ontology (GO) analysis. DEGs of *Pd–*KO versus wild-type in same or different clusters was carried out using Seurat function FindMarkers. The DEGs with adjusted *P* value < 0.01 were assigned as *Pd–KO* increased genes (avg_log2FC >1) or were assigned as *Pd–KO* decreased genes (avg_log2FC <-1). Those DEGs were used for GO biological process analysis with function enrichGO or KEGG pathway analysis using function enrichKEGG in the R package clusterProfiler (v4.10.1) [63]. The top10 pathways of GO biological process analysis or KEGG pathway results were visualized by Seurat DotPlot function.

### 4.24 CytoTRACE2

The normalized expression matrix for *Pdgfrα*+ and adipocyte subtypes was extracted from WT, *Mgp-KO*, and *Pd–KO* integrated data, and analyzed separately using CytoTRACE2 [64] to obtain potency score and potency category predictions. The CytoTRACE score for each cell was then plotted on boxplot. High potency score means less differentiation and earlier progenitor cells.

### 4.25 Pseudotime trajectory construction

Pseudotime trajectories for *Pdgfra*-expressing adipose progenitor cells and adipocytes from WT, *Mgp-*KO and *Pd–*KO, were individually constructed using the R package Monocle3 (v.1.3.7) [65]. The cells were processed with the functions: estimate_size_factors, cluster_cells, learn_graph, and order cells, with default parameters. The root of the trajectory was determined based on the potency score from the CytoTRACE2 result and previously published markers. The cluster with the highest potency score cluster was assigned as the root of the trajectory, and the cells were plotted along the pseudotime. The genes of interest were plotted to visualize their expression along the trajectory.

### 4.26 Data availability

The scRNA-seq data sets of CD45- SVF from inguinal adipose tissue from WT, *Mgp-*KO, *Pd–*KO mice are accessible from GEO database with accession number GSE 296596.

### 4.27 Statistics

Statistical analyses were performed using GraphPad InStat (version 9.0; Graph Pad Software Inc.). Data were analyzed by either unpaired two-tailed Student’s *t* test or one way ANOVA with Tukey’s multiple comparisons test for statistical significance. Data are represented as mean ± SEM. *P* values of less than 0.05 were considered significant, and experiments were repeated a minimum of 3 times.

## CRediT authorship contribution statement

**Li Zhang:** Conceptualization, Data curation, Formal analysis, Methodology, Writing – original draft, Writing – review & editing. **Xinjiang Cai:** Funding acquisition, Methodology, Writing – review & editing. **Xiuju Wu:** Data curation, Writing – review & editing. **Zheng Jing:** Data curation, Methodology. **Yan Zhao:** Data curation, Methodology. **Yucheng Yao:** Funding acquisition, Investigation, Resources. **Kristina I. Boström:** Conceptualization, Funding acquisition, Investigation, Supervision, Writing – original draft, Writing – review & editing.

## Funding

10.13039/100000025National Institutes of Health grant R01HL81397 (KIB), R01HL154548 (KIB), R01NS79353 (YY), R01HL139675 (YY), R01HL162643 (YY), K08HL168147 (XC).

## Declaration of competing interest

The authors declare that they have no known competing financial interests or personal relationships that could have appeared to influence the work reported in this paper.

## Data Availability

Data will be made available on request.
