# Supplementary material for: Regulating the cell differentiation trajectory of progenitor cells in adipose tissue fibrosis
Source: Mol Metab. 2025 Aug 6;100:102231. doi: 10.1016/j.molmet.2025.102231 (PMC12396487; doi:10.1016/j.molmet.2025.102231)
Supplement: Multimedia component 1 [file mmc1.docx]

**Supplemental Table 1: KEY RESOURCES**

| **REAGENT or RESOURCE** | **SOURCE** | **IDENTIFIER** |
| --- | --- | --- |
| **Antibodies - Immunofluorescence** | | |
| CD31, rat polyclonal | R&D Systems | Cat# AF3628 |
| Fibronectin, rabbit polyclonal | Sigma-Aldrich | Cat# F3648 |
| DPP4, rabbit monoclonal | Abcam | Cat# Ab187048 |
| PDGFRα, goat polyclonal | R&D Systems | Cat# AF1062 |
| Perilipin-1, rabbit polyclonal | Cell Signaling Technology | Cat# 9349S |
| **Antibodies - FACS** | | |
| Icam1, rat monoclonal, Alexa Fluor™ 647 | Thermo Fisher | Cat# A15397 |
| Mouse DPPIV/CD26, rat monoclonal, Alexa Fluor® 594 | R&D Systems | Cat# FAB9541T |
| CD31, goat polyclonal, Allophycocyanin (APC) | Thermo Fisher | Cat#12-0311-82 |
| CD140a, rat monoclonal, BD Horizon™ BV786 | BD Biosciences | Cat# 740930 |
| CD45, rat monoclonal, APC | Thermo Fisher | Cat# 17-0451-82 |
| **Antibodies - Immunoblotting** | | |
| COL1A1, rabbit Monoclonal | Abcam | Cat# ab255809 |
| TGF β Receptor I, rabbit polyclonal | Sigma-Aldrich | Cat# SAB4502958 |
| Phospho-SMAD3, rabbit monoclonal | Cell Signaling Technology | Cat# 9520S |
| Phospho-SMAD2, rabbit monoclonal |  | Cat# 3108S |
| SMAD2/3 (D7G7) XP®, rabbit monoclonal |  | Cat# 8685S |
| TGF-β, rabbit polyclonal |  | Cat# 3711S |
| PPARγ, rabbit monoclonal |  | Cat# 2435 |
| Phospho-HSL, rabbit polyclonal |  | Cat# 4137 |
| HSL, rabbit polyclonal |  | Cat# 4107 |
| Phospho-SMAD1/5, rabbit monoclonal |  | Cat# 9516 |
| GAPDH, rabbit monoclonal |  | Cat# 2118 |
| SMAD4, rabbit monoclonal |  | Cat# 38454 |
| Total SMAD | Santa Cruz Biotechnology | Cat# sc-7153 |
| **Secondary Antibodies and DAPI** | | |
| 4’,6-diamidino-2-phenylindole (DAPI) | Sigma-Aldrich | Cat# D9542 |
| Chicken anti-goat secondary antibodies, Alexa Fluor® 594 | Invitrogen | Cat# A-21468 |
| Chicken anti-rabbit secondary antibodies, Alexa Fluor® 488 | Invitrogen | Cat# A-21441 |
| **Cell line, Medium, and Chemicals** |  |  |
| C3H10T1/2 cells | ATCC^TM^ | Cat# CCL-226 |

| DMEM, high glucose | Gibco | Cat# 11965118 |
| --- | --- | --- |
| 3,3′,5-Triiodo-L-thyronine sodium salt | Sigma-Aldrich | Cat# T6397 |
| 3-Isobutyl-1-methylxanthine |  | Cat# I7018 |
| Dexamethasone |  | Cat# D4902 |
| Indomethacin |  | Cat# I7378 |
| Insulin from bovine pancreas |  | Cat# I5500 |
| Sitagliptin |  | Cat# SML3205 |
| SB431542, TGF-β RI Kinase Inhibitor VI |  | Cat# 616464-5mg |
| LY294002 (DMSO solution) | Abcam | Cat# ab146593 |
| Recombinant Mouse TGF-beta 1 Protein | R&D Systems | Cat# 7666-MB/CF |
| Antibiotic-Antimycotic (100x) | Gibco | Cat# 15240062 |
| **Immunoblotting** | | |
| RIPA Lysis and Extraction Buffer | Thermo Scientific™ | Cat# 89900 |
| NuPAGE™ MES SDS Running Buffer (20X) | Invitrogen™ | Cat# NP000202 |
| NuPAGE™ LDS Sample Buffer (4X) |  | Cat# NP0007 |
| NuPAGE™ Sample Reducing Agent (10X) |  | Cat# NP0009 |
| NuPAGE™ Transfer Buffer (20X) |  | Cat# NP00061 |
| Nitrocellulose Membrane, 0.45 µm |  | Cat# 88018 |
| NuPAGE™ Bis-Tris Mini Protein Gels, 10%, 1.0–1.5 mm |  | Cat# NP0301BOX |
| PageRuler™ Prestained Protein Ladder, 10 to 180 kDa | Thermo Scientific™ | Cat# 26616 |
| PhosSTOP™, phosphatase inhibitor | Roche | Cat# 04906837001 |
| cOmplete™, Mini Protease Inhibitor Cocktail |  | Cat# 11836153001 |
| **Other Chemicals** | | |
| Antigen Unmasking Solution | Vector Laboratories | Cat# H-3300-250 |
| 2-Mercaptoethanol | Gibco | Cat# 21985023 |
| Buffer EL (Erythrocyte Lysis Buffer) | Qiagen | Cat# 79217 |
| **Virus Strains and Transfection Reagents** | | |
| Control shRNA Lentiviral particles | Santa Cruz Biotechnology | Cat# Sc108080 |
| MGP shRNA Lentiviral particles |  | Cat# sc-44627-V |
| Polybrene |  | Cat# SC-134220 |
| Puromycin | Sigma-Aldrich | Cat# P9620 |
| **Commercial Assays** | | |

| Corning® 40μm Cell Strainer, Sterile | Corning | Cat# 431750 |
| --- | --- | --- |
| Total Collagen Assay Kit | Abcam | Cat# ab222942 |
| Picro Sirius Red Stain Kit |  | Cat# ab150681 |
| Trichrome Stain Kit |  | Cat# ab150686 |
| RNeasy Mini Kit | Qiagen | Cat# 74106 |
| High-Capacity cDNA Reverse Transcription Kit | Thermo Fisher | Cat# 4374967 |
| TaqMan™ Universal Master Mix II, With UNG |  | Cat# 4440039 |
| **Deposited Data** |  |  |
|  | GEO | TBD |
| **Software and Algorithms** | | |

| GraphPad Prism | GraphPad Software Inc. | https://www.graphpad.com/scientific-software/prism/ |
| --- | --- | --- |
| Cell Ranger v6.1.1 | 10x Genomics | https://www.10xgenomics.com/support/software/cell-ranger/latest/release-notes/cr-release-notes |
| Seurat v5.0.3 |  | https://satijalab.org/seurat/ |
| Monocle3 v1.3.7 |  | https://github.com/cole-trapnell-lab/monocle3/blob/master/NEWS.md |
| g:Profiler |  | https://biit.cs.ut.ee/gprofiler/gost |
