# Supplementary material for: Regulating the cell differentiation trajectory of progenitor cells in adipose tissue fibrosis
Source: Mol Metab. 2025 Aug 6;100:102231. doi: 10.1016/j.molmet.2025.102231 (PMC12396487; doi:10.1016/j.molmet.2025.102231)
Supplement: Multimedia component 2 [file mmc2.docx]

**Supplemental Table 2. Primers for qRT-PCR and genotyping (mouse)**

| **Primer** | **Oligonucleotides / Cat#** | **Experiment** |
| --- | --- | --- |
| Gapdh | Mm99999915_g1 (Thermo Fisher Scientific) | RT-qPCR |
| Mgp | Mm01615497_m1 | RT-qPCR |
| Alk5 | Mm00436964_m1 | RT-qPCR |
| Col1a1 | Mm00801666_g1 | RT-qPCR |
| Pdgfra | Mm00440701_m1 | RT-qPCR |
| Adipoq | Mm00456425_m1 | RT-qPCR |
| Tgfβ1 | Mm01178820_m1 | RT-qPCR |
| Tnfα | Mm00443258_m1 | RT-qPCR |
| Il1β | Mm00434228_m1 | RT-qPCR |
| Ccl2/Mcp-1 | Mm99999056_m1 | RT-qPCR |
| Ifnγ | Mm01168134_m1 | RT-qPCR |
| Il6 | [Mm00446190_m1](https://www.thermofisher.com/taqman-gene-expression/product/Mm00446190_m1?CID=&ICID=&subtype=) | RT-qPCR |
| *Adgre1* (F4/80) | [Mm00802529_m1](https://www.thermofisher.com/taqman-gene-expression/product/Mm00802529_m1?CID=&ICID=&subtype=) | RT-qPCR |
| Mgp_WT-F | GCCACAATTTCTGCATCCTGC | Genotyping |
| Mgp_WT-R | CGGGAAAGATGAGGAAGAAGGG | Genotyping |
| Mgp-mutant-F | TGCCTGAAGTAGCGGTTGTA | Genotyping |
| Mgp-mutant-R | TGAATGAACTGCAGGACGAGG | Genotyping |
| Mgp^flox^-F | CTTAGAAACTGCTGGGTCACGTAGC | Genotyping |
| Mgp^flox^-R | CTTATTTGTCACACGCTGTCACTGTC | Genotyping |
| Pdgfra-Cre Tg-F | TCAGCCTTAAGCTGGGACAT | Genotyping |
| Pdgfra-Cre Tg-R | ATGTTTAGCTGGCCCAAATG | Genotyping |
