# Supplementary material for: Regulating the cell differentiation trajectory of progenitor cells in adipose tissue fibrosis
Source: Mol Metab. 2025 Aug 6;100:102231. doi: 10.1016/j.molmet.2025.102231 (PMC12396487; doi:10.1016/j.molmet.2025.102231)
Supplement: Multimedia component 3 [file mmc3.docx]

**SUPPLEMENTAL FIGURES**


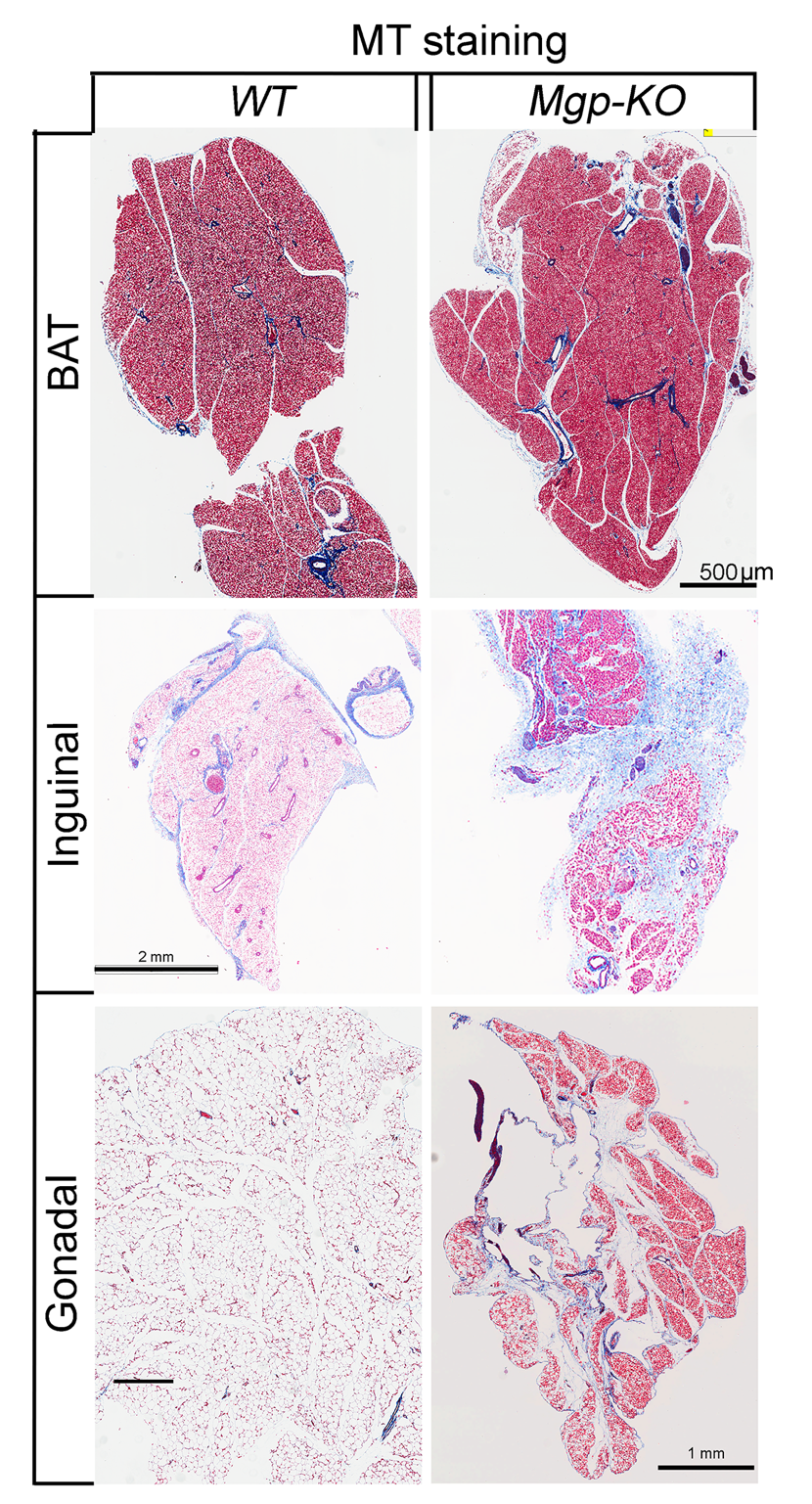


***Supplemental Figure S1***

*Global Mgp deletion in mice enhances adipose fibrosis.*

Masson’s trichrome (MT) staining of adipose tissue from different deposits from wild-type (WT) and *Mgp*-knockout (*Mgp-*KO) mice at 4 weeks of age. Based on the tissue sizes, the scale bars differ: brown adipose tissue (BAT) = 500 µm, inguinal adipose tissue = 2 mm, gonadal adipose tissue = 1 mm.


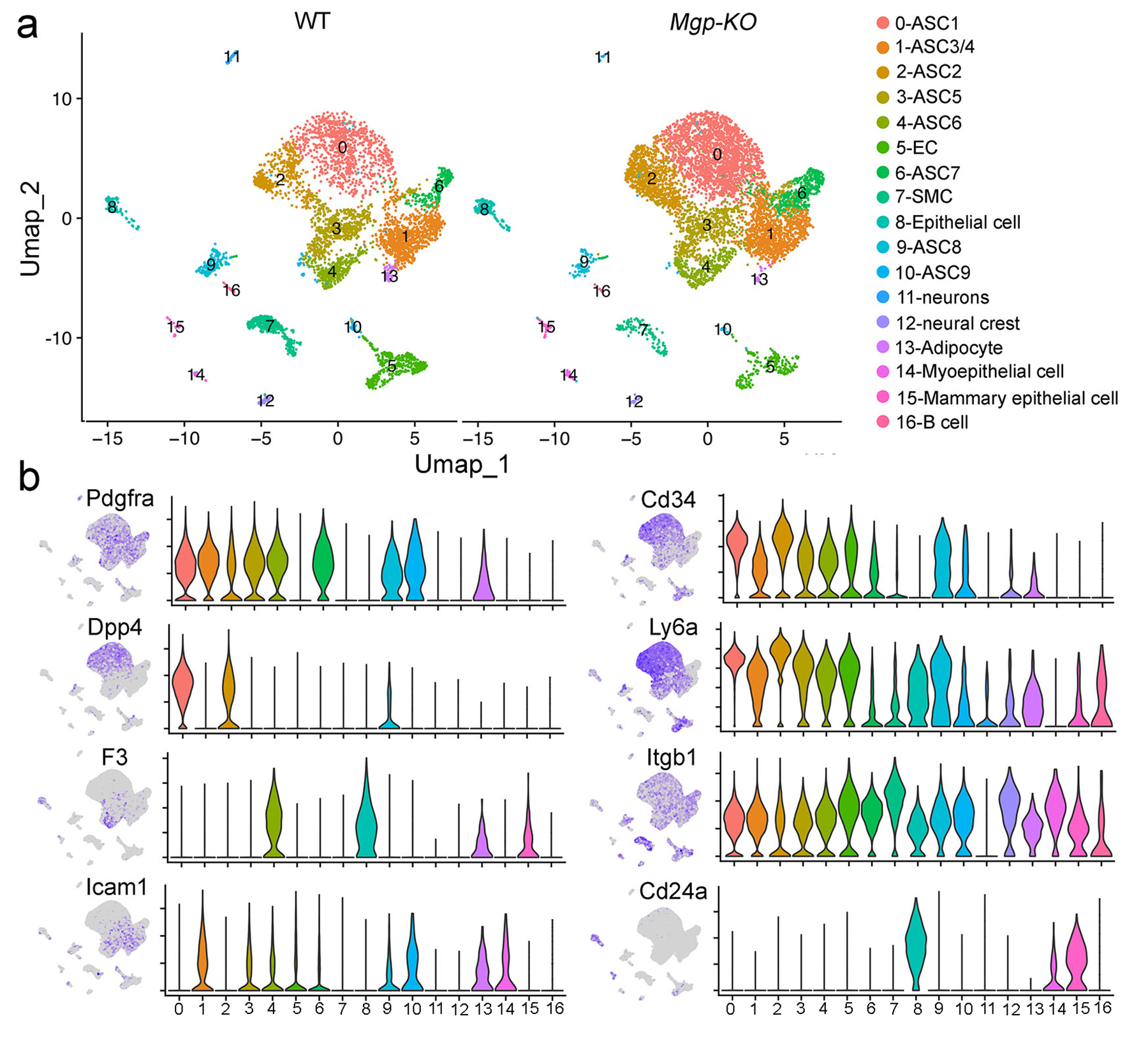


***Supplemental Figure S2***

*Pdgfra-expressing cells include all adipose progenitor cells (APCs).*

**a.** UMAP plot of different cell types of *Cd45*- stromal vascular fraction (SVF) cells from inguinal adipose tissue from wild-type (WT) and *Mgp*-knockout (*Mgp-*KO) mice.

**b.** FeaturePlot and VlnPlot show the expression of cell markers in the different APC clusters, including *Pdgfra*, *Dpp4*, *F3 / Cd142*, *Icam1*, *Ly6a*, Cd34, *Itgab1/ Cd29*, and *Cd24a*.


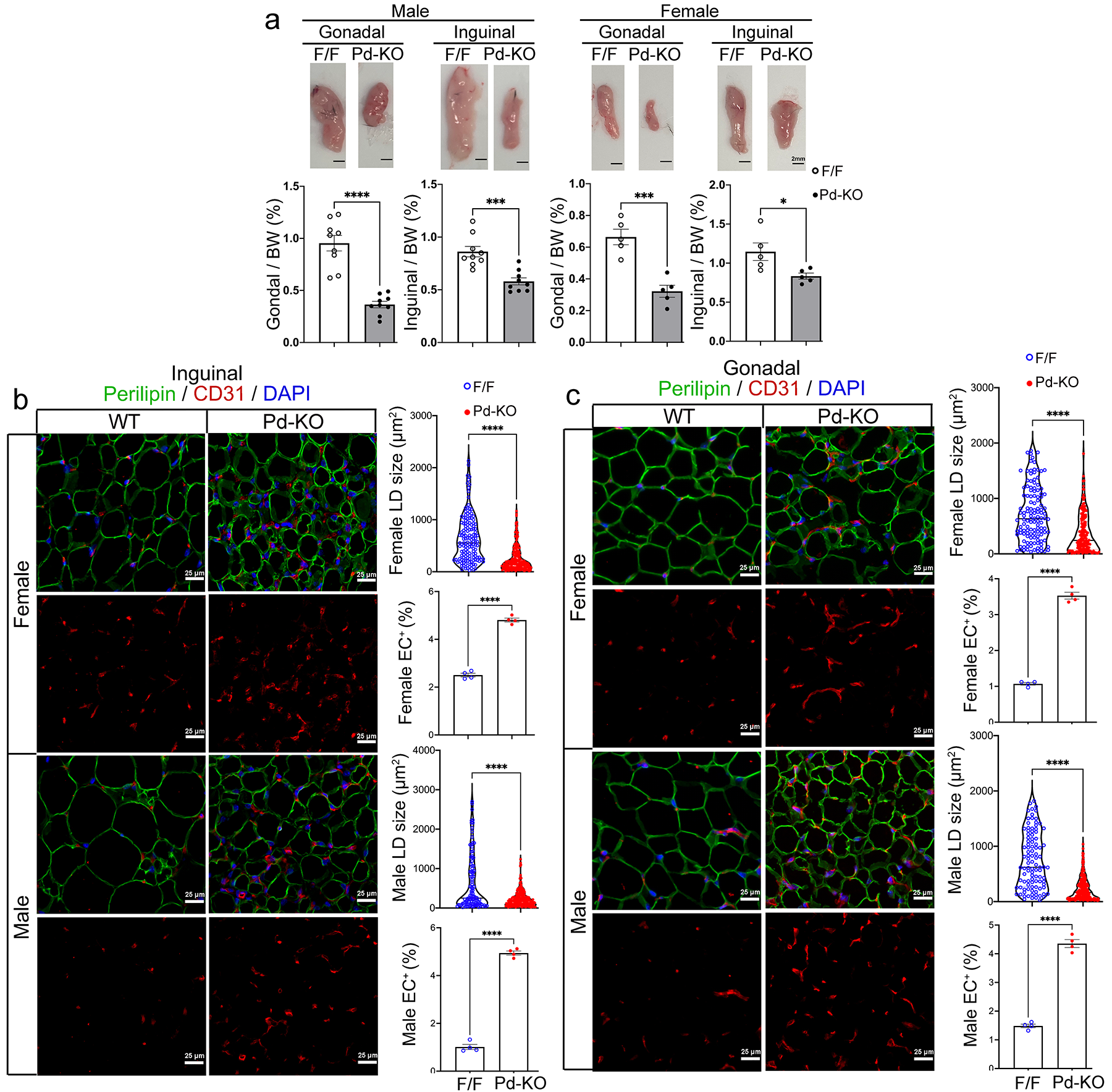


***Supplemental Figure S3***

*White adipose tissue characteristics in Mgp^fl/fl^ (F/F) and Mgp^fl/fl^,Pd^Cre^ (Pd-KO) mice at 4 weeks of age.*

**a.** (Top) Dissected inguinal and gonadal fat pads from male and female F/F and *Pd*-KO mice at 4 weeks of age. (Bottom) Weight of fat pads normalized to body weight (9 male, 5 females per group, respectively).

**b, c.** Immunofluorescence for Perilipin-1 (green, for lipid droplet (LD) size) and CD31 (red) in inguinal (b) and gonadal (c) adipose tissue from F/F and *Pd*-KO mice at 4 weeks of age. DAPI (blue) was used to visualize nuclei. Scale bars, 25 µm. The LD size and microvascular density (CD31-positive area) were quantified by ImageJ (n = 4 mice, 3-4 fields of view per replicate). The LD were smaller in the *Pd*-KO mice compared to F/F controls. The microvascular density was increased in both inguinal and gonadal adipose tissue from *Pd*-KO mice compared to F/F controls.

Data are shown as mean+SEM; One way ANOVA with Tukey’s post hoc tests; *** p<0.001, **** p<0.0001.


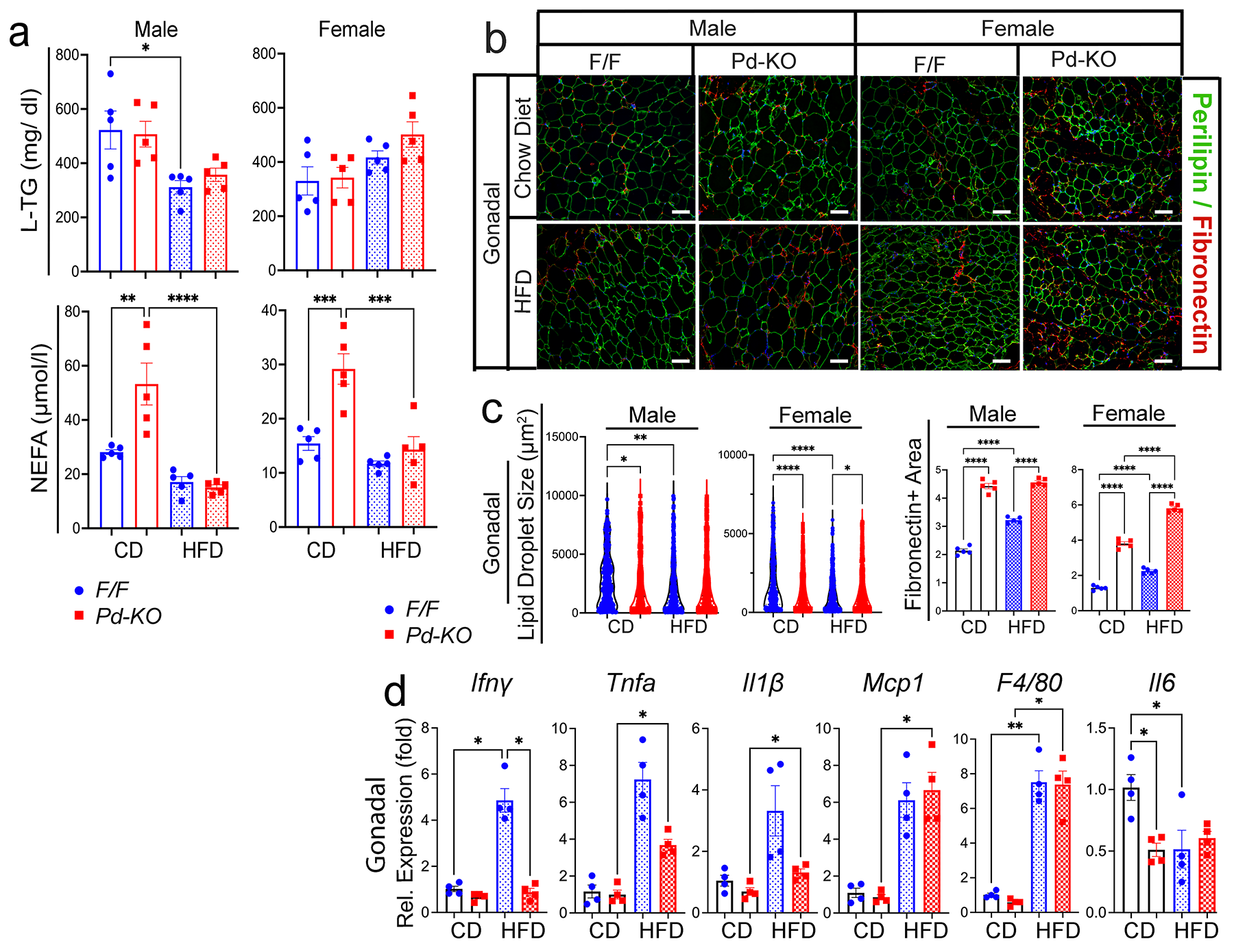


***Supplemental Figure S4***

*Gonadal WAT - Pdgfrα-Cre-guided Mgp deletion enhances adipose fibrosis after high fat feeding*

a. Triglycerides and free fatty acids in serum from F/F and *Pd*-KO mice at 28 weeks (n = 5).

b. Immunofluorescence for Perilipin-1 (green), Fibronectin (red) in gonadal adipose tissue from F/F and *Pd*-KO mice at 28 weeks. DAPI (blue) was used to visualize the nuclei. Bars, 50 µm (n = 5, representative of 3-4 fields of view per replicate).

c. Quantification of Fibronectin positive area and lipid droplet size in Panel b from F/F and *Pd*-KO mice at 28 weeks (n = 5, 3-4 fields of view per replicate).

d. Gene expression of *Ifnγ* (Interferon γ), *Tnfa* (Tumor Necrosis Factor), *Il1β* (Interleukin 1β)*,* *Mcp1* (Monocyte chemoattractant protein-1, also known as *CCL2*)*,* F4/80 (also known as *Adgre1,* a macrophage marker), and *Il6* (Interleukin 6) in gonadal adipose tissue from F/F and *Pd*-KO mice at 28 weeks as determined by qPCR (n = 4 per group).

Data are shown as mean+SEM; One way ANOVA with Tukey’s post hoc tests; ** p<0.01, *** p<0.001, **** p<0.0001.


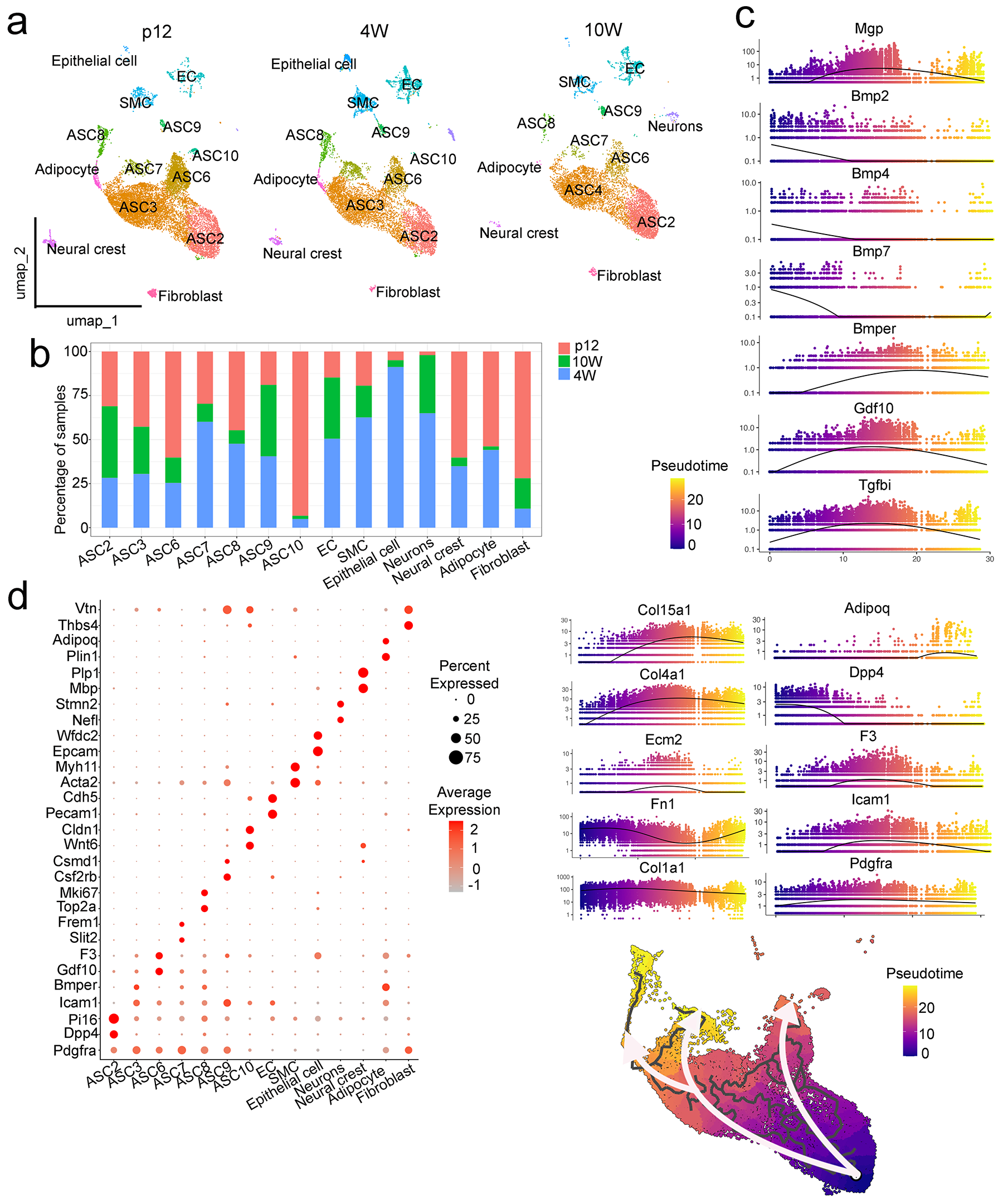


***Supplemental Figure S5***

*Analysis of scRNA-seq data from inguinal adipose tissue from differently aged mice.*

a. UMAP plot of different cell types of *Cd45*- SVF-cells from inguinal adipose tissue from wild-type (WT) mice postnatal day 12 (p12, GSM3717977), aged 4 weeks (4W, GSM8973244), and 10 weeks (10W, GSM3717978).

b. Percent cells in each cell type at different time point including postnatal day 12 (p12, red), 10 weeks (10W, green), and 4 weeks (4W, blue) by ggplot.

c. Marker genes for each cell cluster.

d. Pseudotime plots of TGFβ pathway-related genes (top), extracellular matrix (ECM) genes (mid left), adipose progenitor genes (mid right). UMAP visualization of trajectory analysis of APCs and early adipocytes by Pseudotime (bottom)


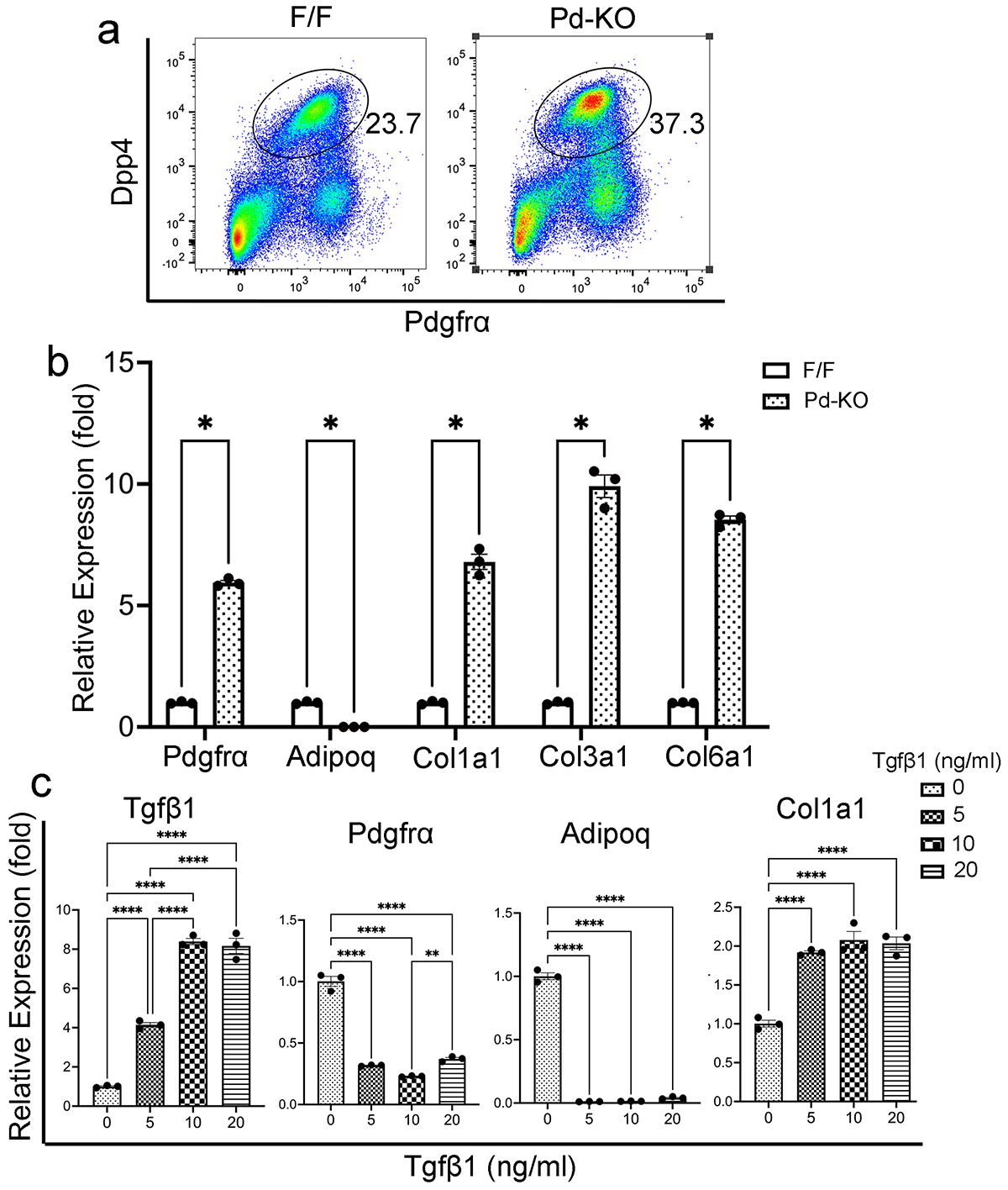


***Supplemental Figure S6***

*PDGFRα+;Dpp4+ cell characteristics in Mgp*^f/f^ (F/F) *and Mgp^fl/fl^,Pd^Cre^ (Pd-KO) mice.*

a. FACS analysis of PDGFR*α*+, DPP4+ double positive cell populations after removal of immune and endothelial cells (n = 3 mice per group), from the inguinal stromal vascular fraction of F/F and *Pd*-KO mice.

b. Expression of *Pdgfra, Adipoq*, *Col1a1, Col3a1, and Col6a1* in isolated PDGFR*α*+;DPP4+ cell populations from *Mgp*^f/f^ and *Pd*-KO mice, as determined by qPCR (n = 3).

c. PDGFRa⁺;Dpp4⁺ cells isolated from F/F mice were induced to undergo adipogenesis and treated with varying concentrations of TGFβ1 (0–20 ng/ml). Expression of *Tgfb1, Pdgfrα, Adipoq,* and *Col1a1* of PDGFRα⁺;Dpp4⁺ cells after 12 days of treatment with different concentrations of TGFβ1 (0-20 ng/ml), as determined by qPCR.

Data are shown as mean+SEM; One way ANOVA, * p<0.05, ** p<0.01, **** p<0.0001.


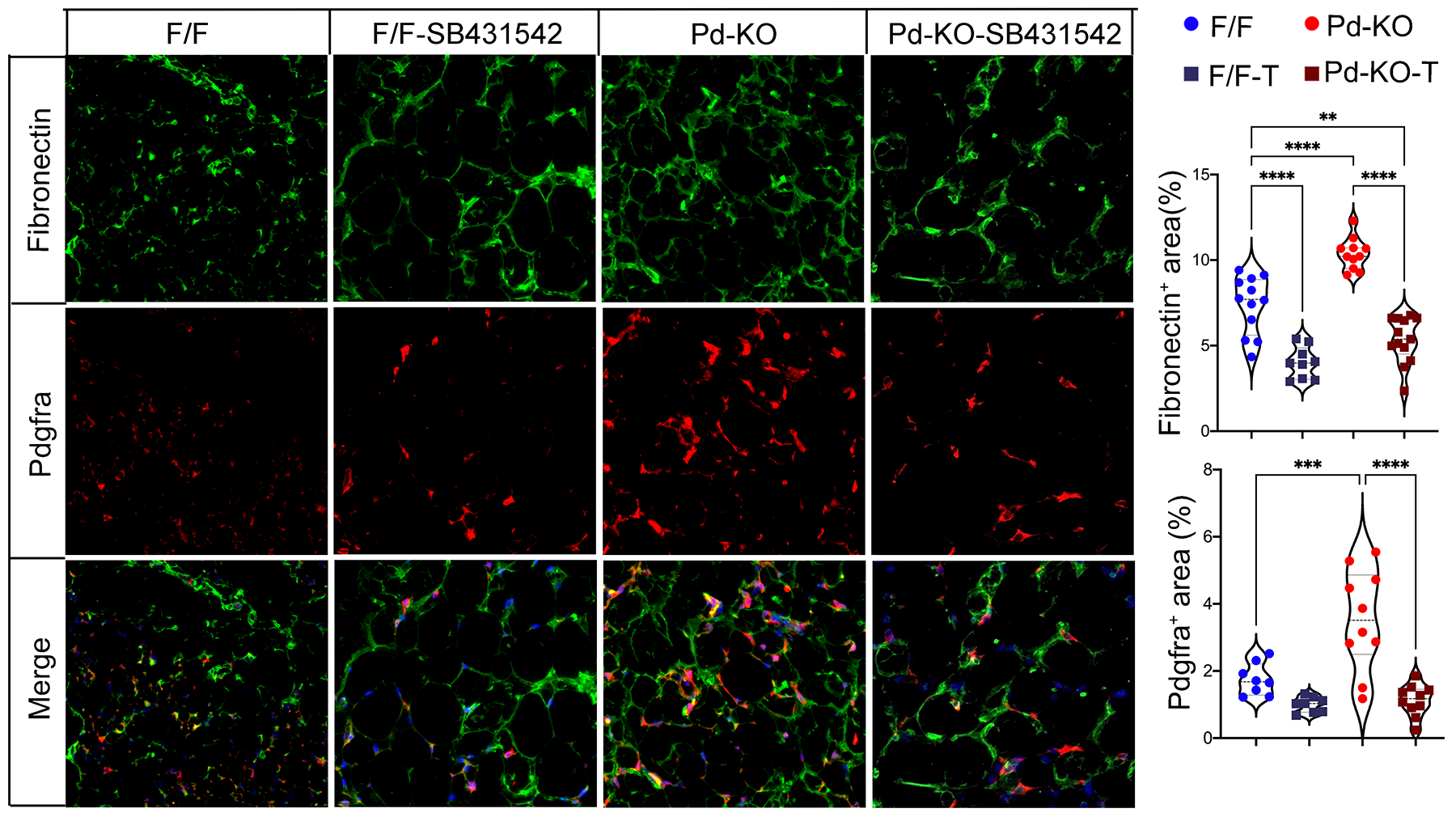


***Supplemental Figure S7***

*ALK5 inhibition limits adipose fibrosis caused by Pdgfra Cre-mediated Mgp deletion.*

(Left) Immunofluorescence for Fibronectin (green) and PDGFRα (red) in inguinal adipose tissue from *Mgp^f^*^/f^ (F/F) and *Mgp^fl/fl^,Pd^Cre^ (Pd-*KO*)* mice after treatment with SB431542 in 10% DMSO or 10% DMSO control. (Right) Quantification of immunofluorescence for Fibronectin and PDGFRα (random selection of 3 - 4 areas from each paraffin-fixed section; n = 3).

Data are shown as mean+SEM; One way ANOVA, ** p<0.01, *** p<0.001, **** p<0.0001.


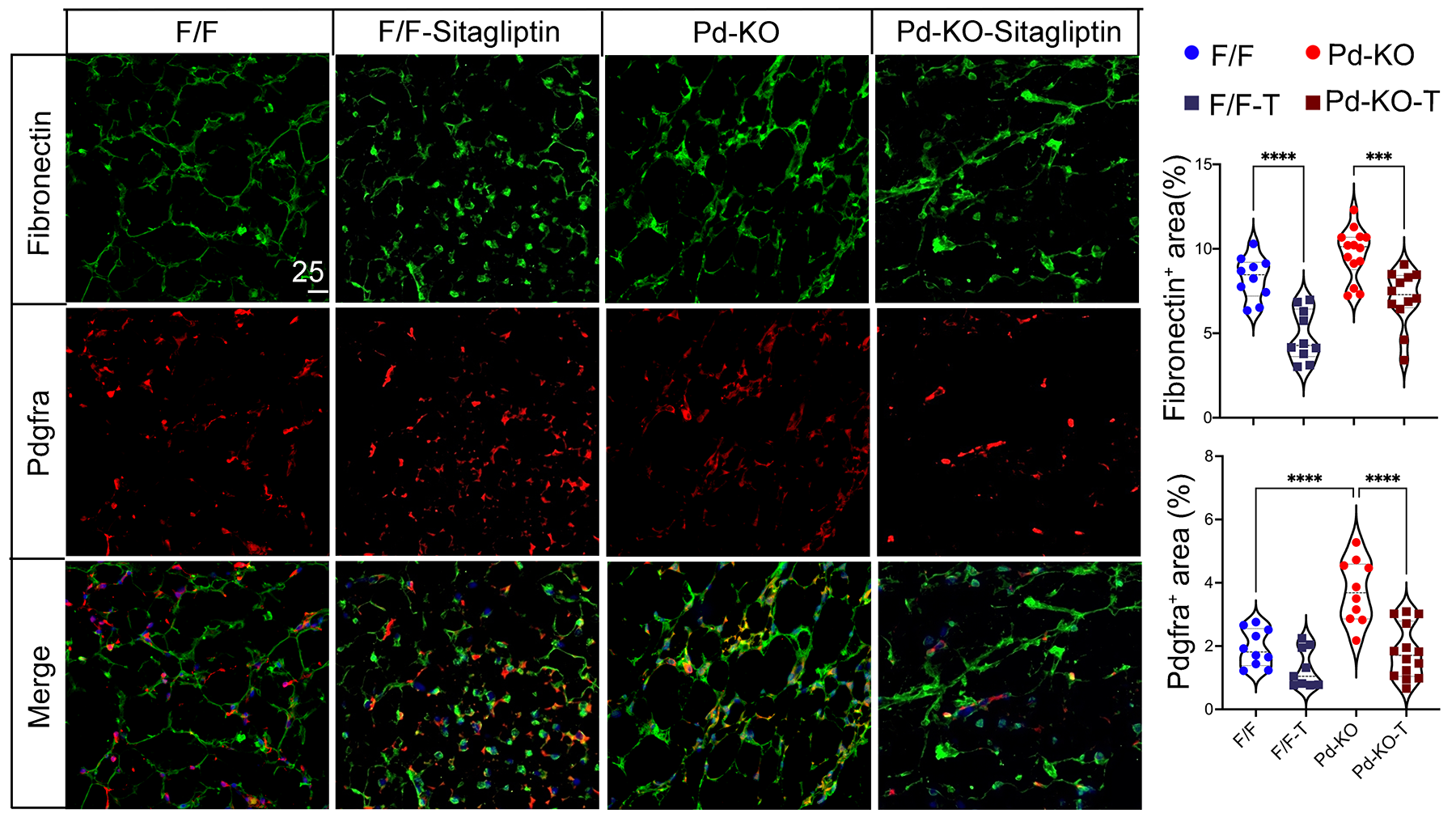


***Supplemental Figure S8***

*DPP4 inhibition limits adipose fibrosis caused by Pdgfra Cre-mediated Mgp deletion.*

(Left) Immunofluorescence for Fibronectin (green) and PDGFRα (red) in inguinal adipose tissue from *Mgp^f^*^/f^ (F/F) and *Mgp^fl/fl^,Pd^Cre^ (Pd-*KO*)* mice after treatment with sitagliptin in 5% DMSO or 5% DMSO control. (Right) Quantification of immunofluorescence for Fibronectin and PDGFRα (random selection of 3 - 4 areas from each paraffin-fixed section; n = 3).

Data are shown as mean+SEM; One way ANOVA, ** p<0.01, *** p<0.001, **** p<0.0001.


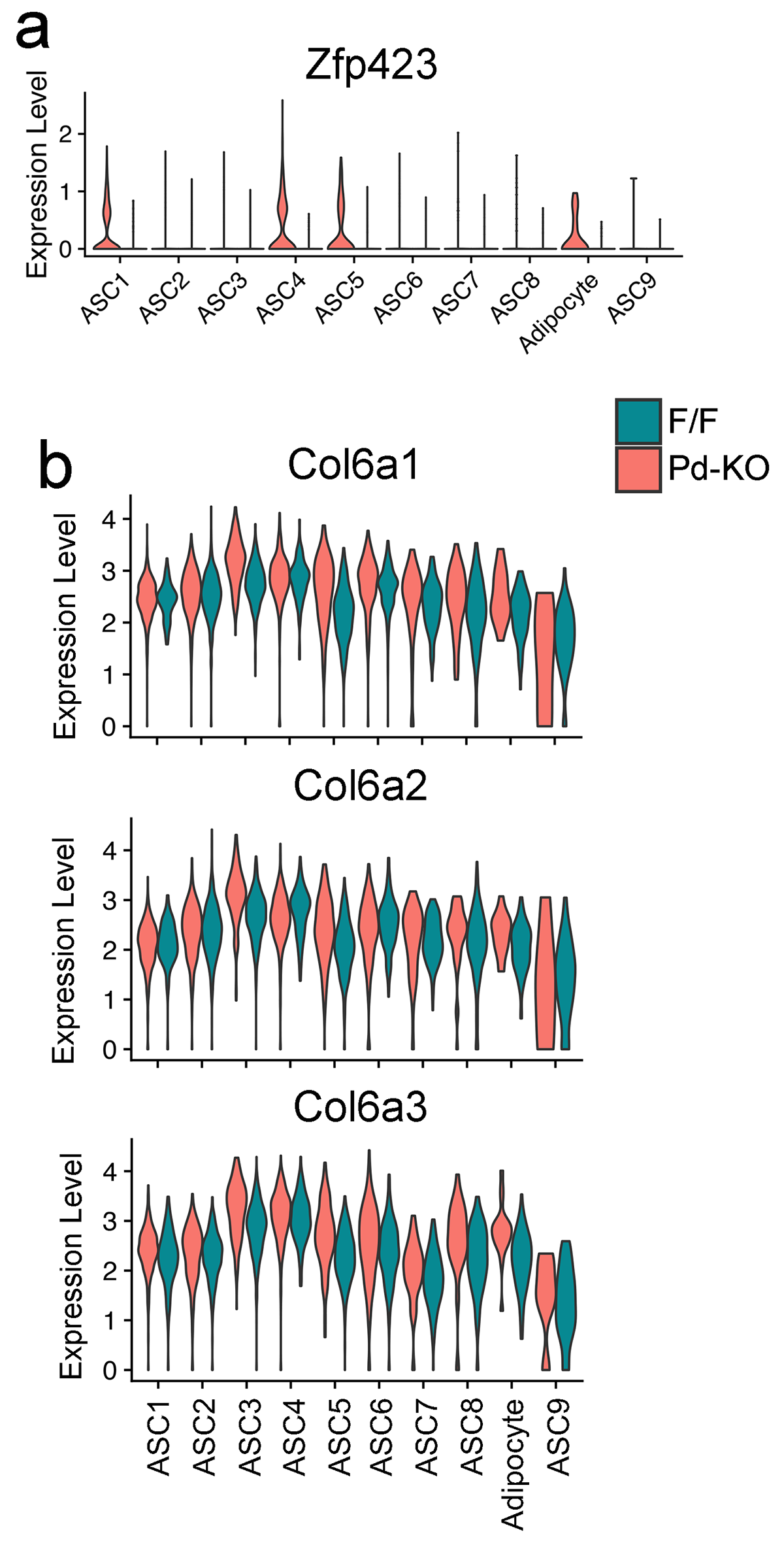


***Supplemental Figure S9***

a. Expression of *Zfp423* in scRNA-seq of *Pdgfra*-expressing ASC clusters and early adipocytes in F/F and Pd-KO white adipose tissue.

b. Expression of *Col6a1, Col6a2* and *Col6a3* in scRNA-seq of *Pdgfra*-expressing ASC clusters and early adipocytes in F/F and Pd-KO white adipose tissue.


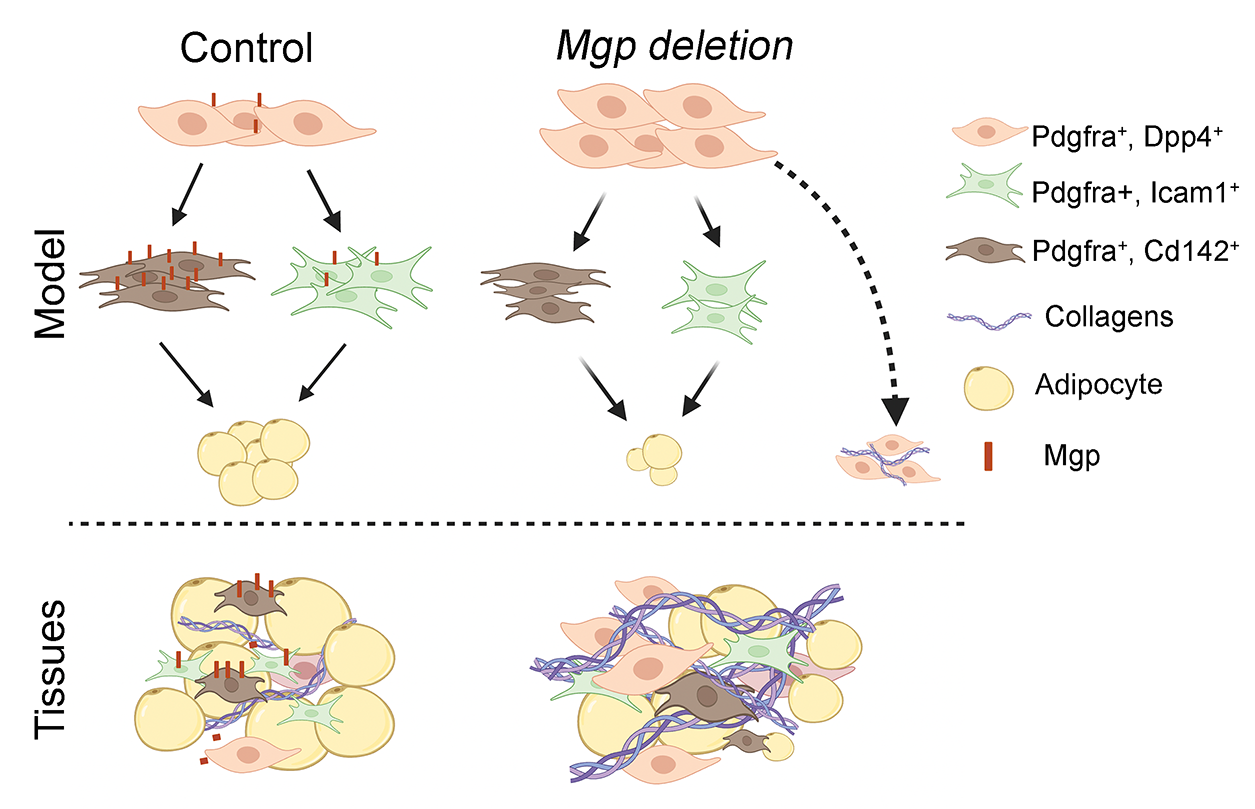


***Supplemental Figure S10***

*Working model of matrix Gla protein (MGP)-regulated cell trajectory of* adipose progenitor cells (*APCs) for developiong adipose fibrosis.*

(Left) *Pdgfra+;Dpp4+* APCs differentiate to *Pdgfra+;Icam1+* pre-adipocytes, undergo differentiate to adipocytes and form normal white adipose tissue. (Right) After *Mgp* deletion*, Pdgfra+;Dpp4+* APCs divert to a different cellular trajectory related to fibrogenesis, resulting in adipose fibrosis. Pro-fibrotic factors may trigger this pathway by modulating MGP or counterparts. (*Cartoon model by BioRender*.)
